# Supplementary material for: Single cell genomics reveals plastid-lacking Picozoa are close relatives of red algae
Source: Nat Commun. 2021 Nov 17;12:6651. doi: 10.1038/s41467-021-26918-0 (PMC8599508; doi:10.1038/s41467-021-26918-0)
Supplement: Supplementary file 1 — Supplementary Information [file 41467_2021_26918_MOESM1_ESM.pdf]

Supplementary Information
for
Single cell genomics reveals plastid-lacking Picozoa are close relatives of red algae

Max E. Schön, Vasily V. Zlatogursky, Rohan P. Singh, Camille Poirier, Susanne Wilken, Varsha Mathur, Jürgen F. H. Strassert, Jarone Pinhassi, Alexandra Z. Worden, Patrick J. Keeling, Thijs J. G. Ettema, Jeremy G. Wideman, Fabien Burki*

List of Figures

1 Maximum Likelihood tree of the 18S rRNA gene. 6

2 Combined relative abundance of all Picozoa OTUs identified in the Tara Oceans metabarcoding data. 7

3 Maximum likelihood tree of 794 eukaryotic species. 8

4 Support for several groupings as estimated in different trees with increasing number of fast-evolving sites removed. 9

5 Maximum likelihood tree of 67 eukaryotic species showing the position of Picozoa. 9

6 Multi-species coalescent species tree reconstructed with ASTRAL-III. 10

7 Maximum likelihood tree of 67 eukaryotic species showing the position of Picozoa. 11

8 Maximum likelihood tree of 67 eukaryotic species showing the position of Picozoa. 12

9 Complete and near complete mitochondrial genomes assembled from diverse picozoan SAGs. 13

10 Number of inferred lateral gene transfers (LGT) across a selection of 33 species. 14

11 Maximum Likelihood tree of the 18S rRNA gene from the individual SAG assemblies and an extended number of reference sequences from Picozoa and other major eukaryotic groups from the PR2 database. 15

12 Heatmap showing pairwise ANI for 43 initial picozoan SAGs as estimated with FastANI. 16

13 Boxplots of different BUSCO categories (Missing, Complete, Fragmented and Duplicated) for all selected SAGs/Co-SAGs. 16

14 Contamination estimate for each of the 17 final SAGs/Co-SAGs. 17

15 Maximum likelihood tree of eukaryotic species showing the position of Picozoa. . 18

16 Bayesian phylogenetic tree made using PhyloBayes. 19

17 Bayesian phylogenetic tree made using PhyloBayes. 20

List of Tables

1 Additional genomes added to the Phylogenomic dataset 3
2 Results from the AU and other topology tests performed with IQ-TREE 4
3 EGT and LGT results for 33 selected species/groups 5

Supplementary Table 1: Additional genomes added to the Phylogenomic dataset

Ancyromonas sigmoides	CRuMs	https://academic.oup.com/gbe/article/10/2/427/4817507
Diphylleia rotans	CRuMs	https://academic.oup.com/gbe/article/10/2/427/4817507
Fabomonas tropica	CRuMs	https://academic.oup.com/gbe/article/10/2/427/4817507
Nutomonas longa	CRuMs	https://www.sciencedirect.com/science/article/pii/S0960982215008878
Rigifila ramosa	CRuMs	https://academic.oup.com/gbe/article/10/2/427/4817507
Palpitomonas bilix	Cryptista	
Roombia truncata	Cryptista	
Goniomonas avonlea	Cryptomonads	https://bmcbiol.biomedcentral.com/articles/10.1186/s12915-018-0593-5
Cyanophora paradoxa	Glaucophyta	https://academic.oup.com/dnaresearch/article/26/4/287/5490643
		http://cyanophora.rutgers.edu/cyanophora_v2018/
Hemimastix kukwesjijk	Hemimastigophora	https://www.nature.com/articles/s41586-018-0708-8
		https://datadryad.org/stash/dataset/doi:10.5061/dryad.n5g39d7
Spironema sp BW2	Hemimastigophora	https://www.nature.com/articles/s41586-018-0708-8
Andalucia godoyi	Jakobids	https://bmcbiol.biomedcentral.com/articles/10.1186/s12915-020-0741-6
Gefionella okellyi	Malawimonads	https://royalsocietypublishing.org/doi/10.1098/rsos.171707
Carpediemonas membranifera	Metamonada	https://www.nature.com/articles/s41559-017-0092
Chilomastix cuspidata	Metamonada	https://www.nature.com/articles/s41559-017-0092
Dysnectes brevis	Metamonada	https://www.nature.com/articles/s41559-017-0092
Rhodelphis limneticus	Rhodelphidia	https://www.nature.com/articles/s41586-019-1398-6
		https://datadryad.org/stash/dataset/doi:10.5061/dryad.tr6d8q2
Rhodelphis marinus	Rhodelphidia	https://www.nature.com/articles/s41586-019-1398-6
		https://datadryad.org/stash/dataset/doi:10.5061/dryad.tr6d8q2
Gracilariopsis chorda	Rhodophyta	https://academic.oup.com/mbe/article/35/8/1869/4982564
Porphyridium purpureum	Rhodophyta	https://www.nature.com/articles/s41467-019-12779-1
		http://porphyra.rutgers.edu/bindex.php

Supplementary Table 2: Results from the AU and other topology tests performed with IQ-TREE

	Tree	logL	deltaL	bp-RELL	p-KH	p-SH	p-WKH	p-WSH	c-ELW	p-AU
1	(Picozoa),(Rhodelphis,Rhodophyta)	-3576102.049	0.0	0.787	0.792	1.0	0.792	1.0	0.786	0.822
2	(Picozoa),(Rhodophyta)	-3576116.962	14.914	0.203	0.208	0.769	0.208	0.607	0.203	0.237
3	(Picozoa),(Rhodelphis)	-3576132.673	30.624	0.0099	0.031	0.628	0.031	0.162	0.0103	0.0268
4	(Picozoa),(Viridiplantae,Glaucophyta)	-3576302.884	200.84	0.0	0.0	0.0	0.0	0.0	2.15e-48	5.52e-55
5	(Picozoa),(Glaucophyta)	-3576394.956	292.91	0.0	0.0	0.0	0.0	0.0	1.26e-75	1.1e-06
6	(Picozoa),(Viridiplantae)	-3576365.604	263.56	0.0	0.0	0.0	0.0	0.0	1.47e-56	9.63e-43
7	(Picozoa),(Archaeplastida)	-3576299.786	197.74	0.0	0.0	0.0	0.0	0.0	4.52e-43	3.55e-56
8	(Picozoa),(Telonemia)	-3576410.74	308.69	0.0	0.0	0.0	0.0	0.0	7.04e-55	2.07e-08
9	(Picozoa),(Telonemia,Rhizaria,Stramenopila,Alveolata)	-3576454.447	352.4	0.0	0.0	0.0	0.0	0.0	5.42e-76	2.45e-07
10	(Picozoa),(Cryptista)	-3576314.922	212.87	0.0	0.0	0.0	0.0	0.0	1.7e-43	8.25e-61
11	(Picozoa,Cryptista),(Rhodophyta,Rhodelphis)	-3576288.706	186.66	0.0	0.0	0.0005	0.0	0.0	1.84e-11	5.49e-79
12	(Picozoa,Cryptista),(Rhodophyta)	-3576564.043	461.99	0.0	0.0	0.0	0.0	0.0	6.27e-118	2.51e-33
13	(Picozoa,Cryptista),(Viridiplantae,Glaucophyta)	-3576349.415	247.37	0.0	0.0	0.0	0.0	0.0	1.78e-35	2.78e-36
14	(Picozoa,Cryptista),(Glaucophyta)	-3576505.322	403.27	0.0	0.0	0.0	0.0	0.0	9.38e-88	1.08e-57
15	(Picozoa,Cryptista),(Viridiplantae)	-3576461.386	359.34	0.0	0.0	0.0	0.0	0.0	9.78e-75	0.00222

Supplementary Table 3: EGT and LGT results for 33 selected species/groups

Taxon	plastid status	EGT	LGT	EGT/LGT Ratio
Rattus norvegicus	no plastid ancestry	0.0	15.0	0.0
Neurospora crassa	no plastid ancestry	0.0	35.0	0.0
Dictyostelium discoideum	no plastid ancestry	1.0	29.0	0.03448275862069
Tetrahymena thermophila	no plastid ancestry	1.0	23.0	0.043478260869565
Thecamonas trahens	no plastid ancestry	1.0	13.0	0.076923076923077
Picozoa		16.0	99.0	0.161616161616162
Goniomonas pacifica	no plastid ancestry	18.0	90.0	0.2
Telonema subtile	no plastid ancestry	15.0	62.0	0.241935483870968
Hematodinium sp SG-2012	plastid loss	10.0	28.0	0.357142857142857
Phytophthora capsici	no plastid ancestry	6.0	15.0	0.4
Rhodelphis	non-photosynthetic plastid	14.0	32.0	0.4375
Spumella bureschii JBL14	non-photosynthetic plastid	31.0	25.0	1.24
Cryptomonas paramecium	non-photosynthetic plastid	34.0	25.0	1.36
Cryptosporidium muris	plastid loss	3.0	2.0	1.5
Alexandrium tamarense	photosynthetic plastid	218.0	93.0	2.34408602150538
Paraphysomonas bandaiensis	non-photosynthetic plastid	12.0	5.0	2.4
Paulinella chromatophora	photosynthetic plastid	160.0	49.0	3.26530612244898
Cyanophora paradoxa	photosynthetic plastid	228.0	55.0	4.14545454545455
Vitrella brassicaformis	photosynthetic plastid	130.0	24.0	5.41666666666667
Emiliania huxleyi	photosynthetic plastid	177.0	32.0	5.53125
Toxoplasma gondii	non-photosynthetic plastid	17.0	3.0	5.66666666666667
Bigelowiella natans	photosynthetic plastid	161.0	23.0	7.0
Guillardia theta	photosynthetic plastid	143.0	20.0	7.15
Polytomella parva	non-photosynthetic plastid	58.0	8.0	7.25
Helicosporidium sp ATCC50920	non-photosynthetic plastid	59.0	8.0	7.375
Galdieria sulphuraria	photosynthetic plastid	168.0	19.0	8.8421052631579
Mallomonas sp CCMP3275	photosynthetic plastid	89.0	9.0	9.88888888888889
Ochromonadales sp CCMP2298	photosynthetic plastid	110.0	10.0	11.0
Leptocylindrus danicus	photosynthetic plastid	151.0	12.0	12.5833333333333
Pedospumella elongata	non-photosynthetic plastid	19.0	1.0	19.0
Dinobryon sp UTEXLB2267	photosynthetic plastid	122.0	4.0	30.5
Chloropicon primus	photosynthetic plastid	225.0	4.0	56.25
Arabidopsis thaliana	photosynthetic plastid	313.0	0.0	inf

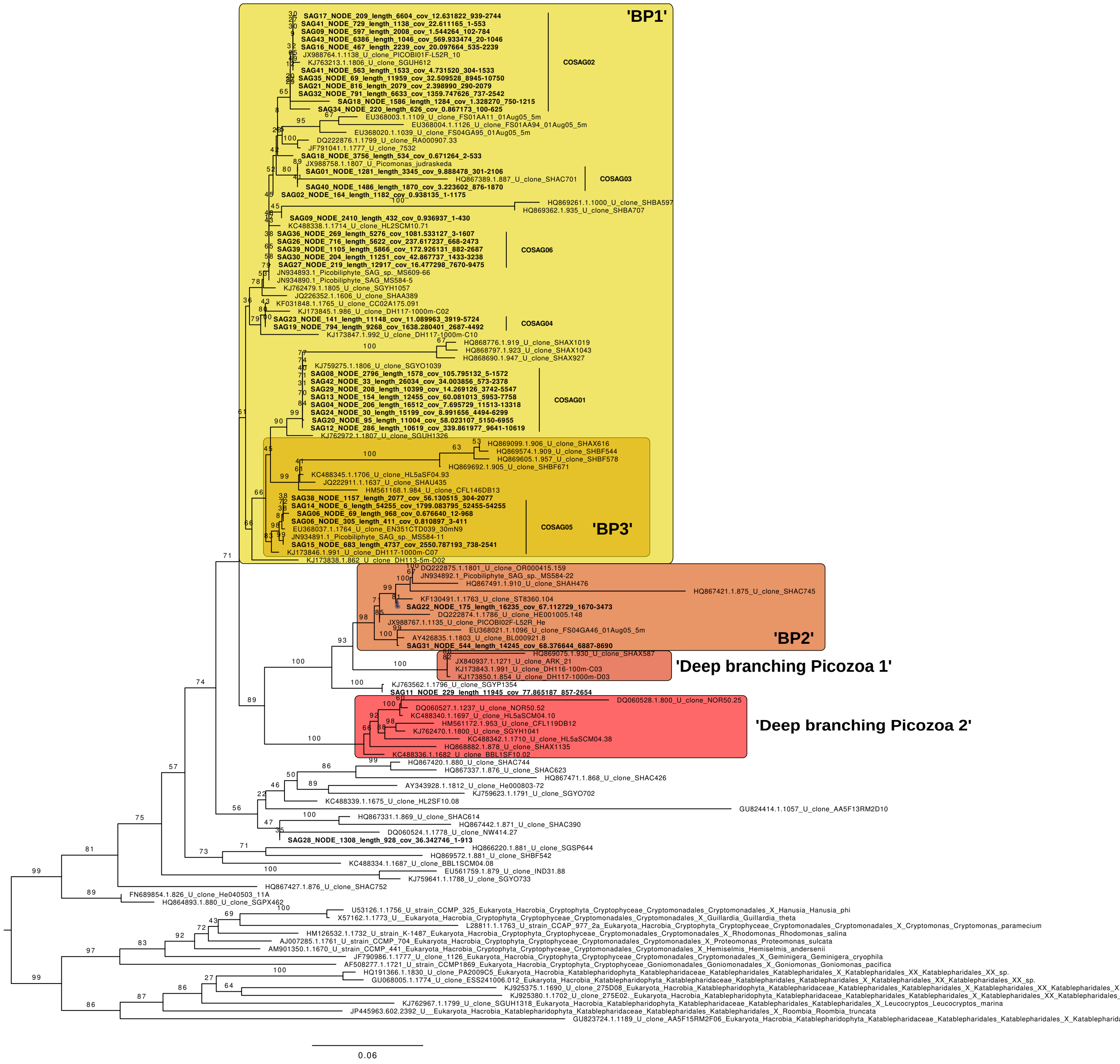

Supplementary Figure 1: Maximum Likelihood tree of the 18S rRNA gene. Sequences from the individual SAG assemblies and a number of reference sequences from Picozoa, cryptophyes katablepharids from the PR2 database. The tree was reconstructed under the model GTR+G.

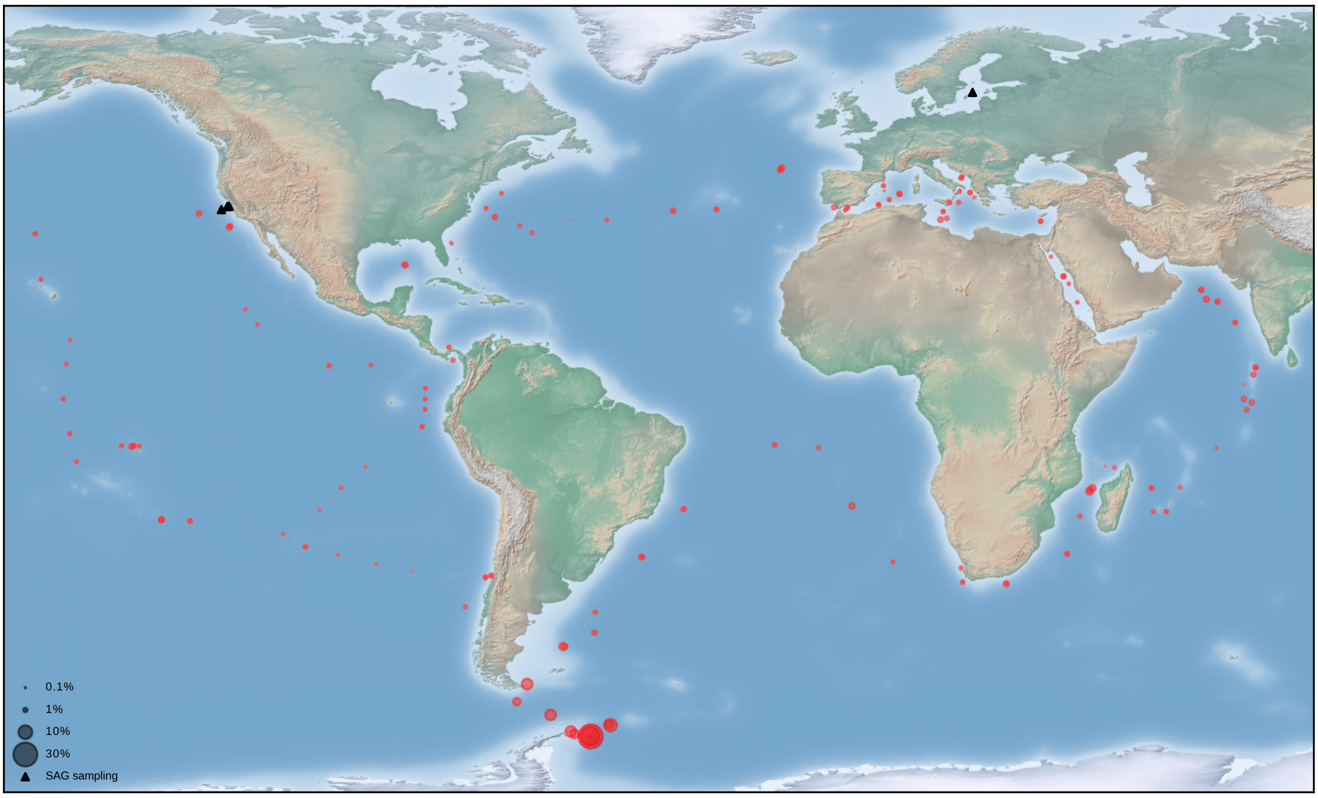

Supplementary Figure 2: Combined relative abundance of all Picozoa OTUs identified in the Tara Oceans metabarcoding data. Abundances are given on a corresponding map of sampling locations. Size of the circles corresponds to relative abundance. Triangles mark the location of single-cell sampling from this study.

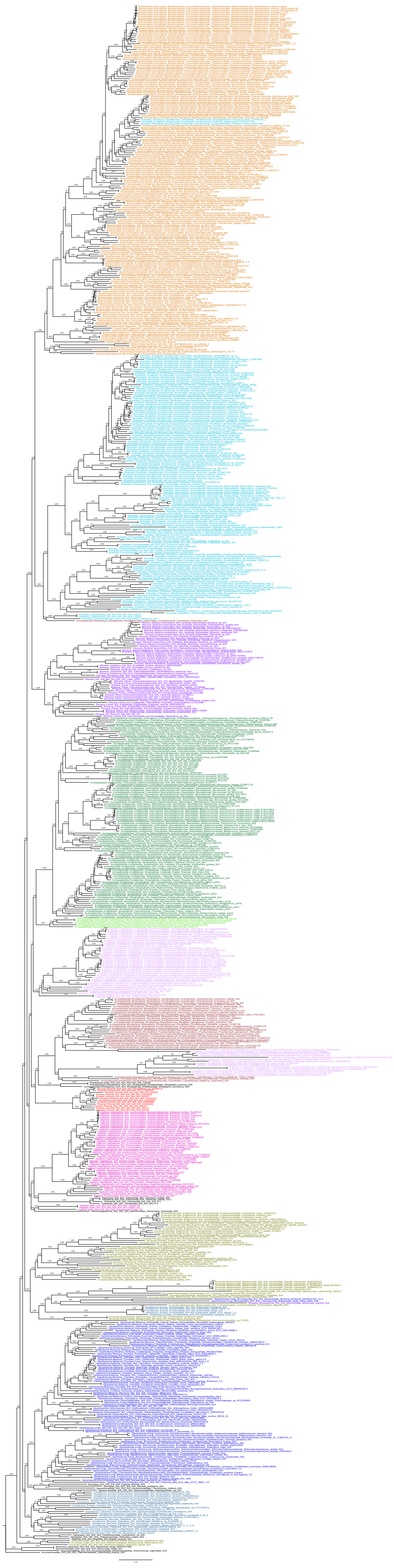

Supplementary Figure 3: Maximum likelihood tree of 794 eukaryotic species. The tree is based on the concatenated alignment of 317 marker genes (filtered with Divvier) and was reconstructed using the site-homogeneous model LG+F+G. Support values correspond to 1000 ultrafast bootstrap replicates.

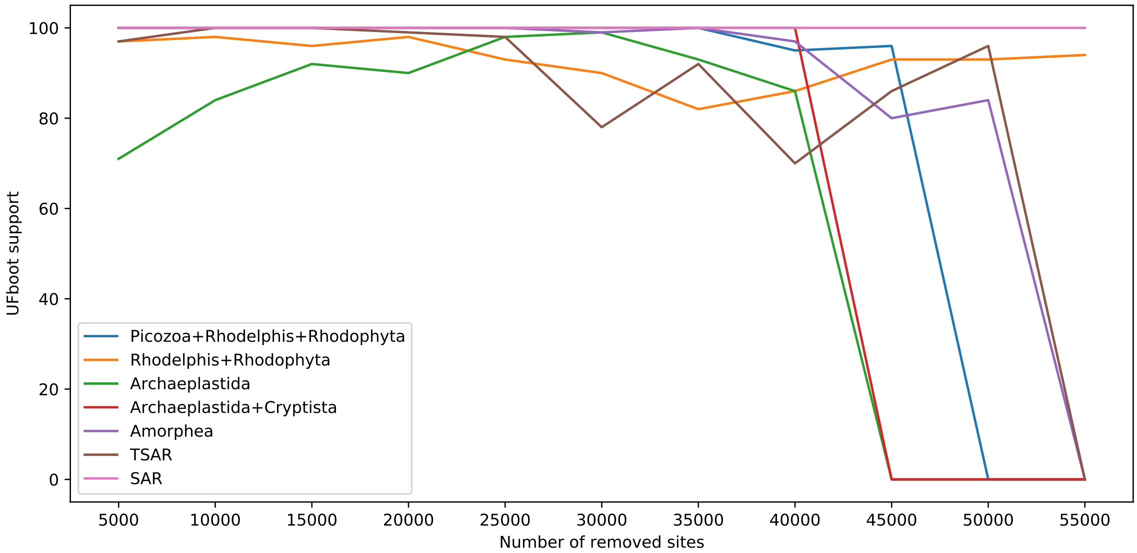

Supplementary Figure 4: Support for several groupings as estimated in different trees with increasing number of fast-evolving sites removed. Initial alignment was the 67-dataset (filtered with BMGE). All trees were reconstructed using the site-heterogeneous model LG+C60+F+G, support values correspond to 1000 ultrafast bootstrap replicates. Source data are provided in a Source Data file.

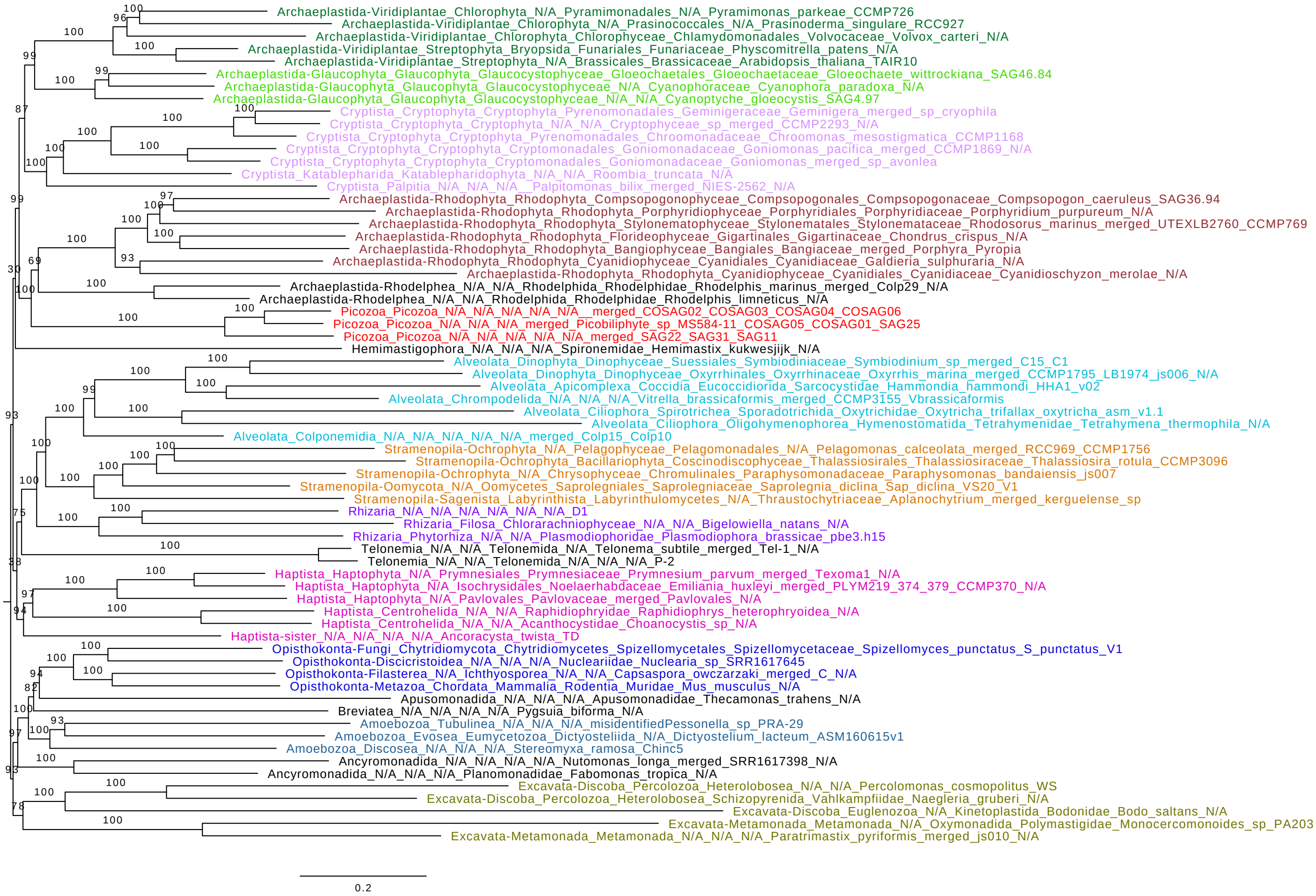

Supplementary Figure 5: Maximum likelihood tree of 67 eukaryotic species showing the position of Picozoa. The tree is based on the concatenated alignment of 317 marker genes, filtered with BMGE and trimmed of the 25% most heterogenous sites according to the chi-square statistic. The tree was reconstructed using the site-heterogeneous model LG+C60+F+G, support values correspond to 1000 ultrafast bootstrap replicates.

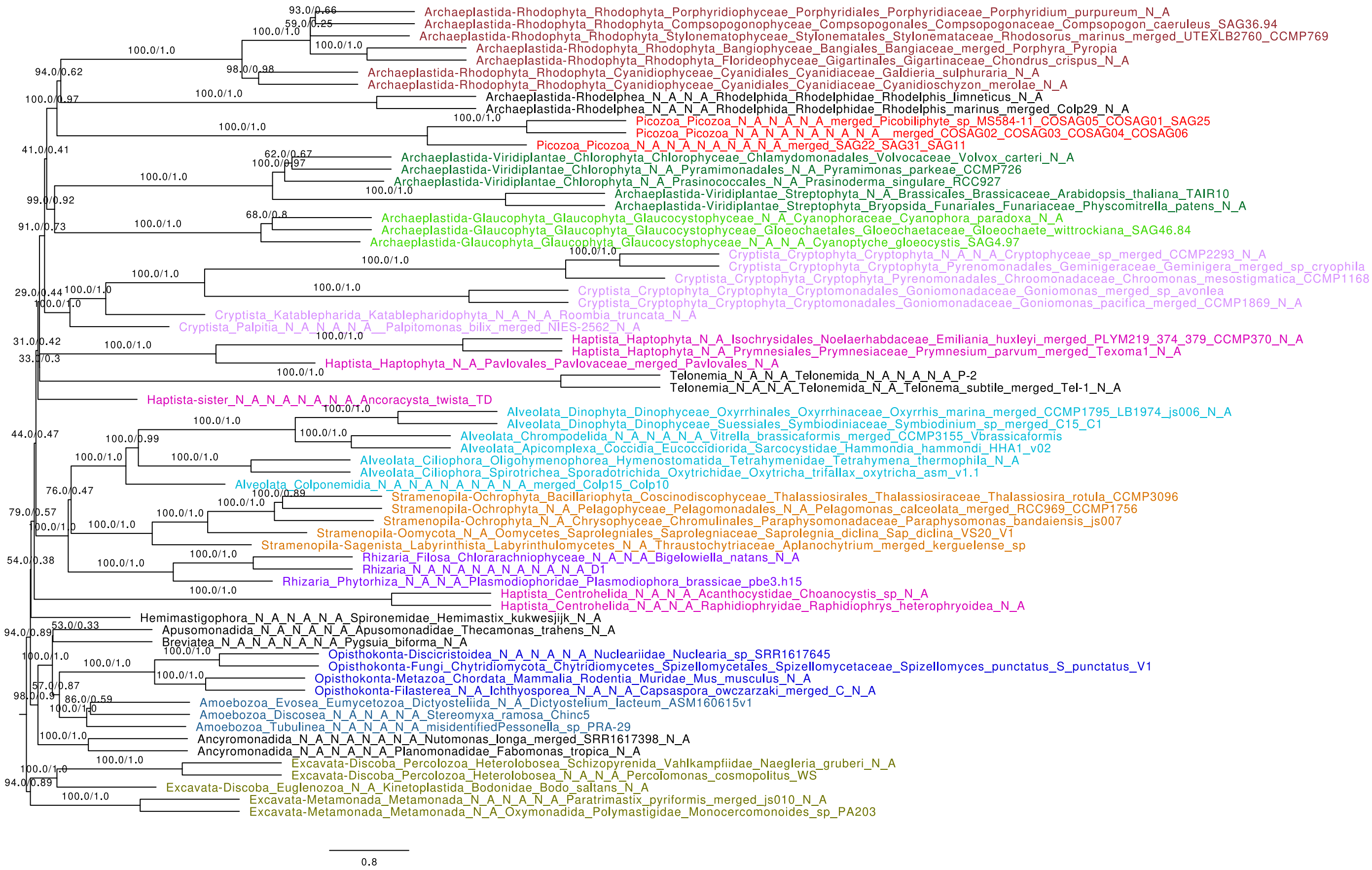

Supplementary Figure 6: Multi-species coalescent species tree reconstructed with ASTRAL-III. Individual single gene trees were inferred for all 317 untrimmed alignments from the 67-taxa dataset, using automatic model selection and 1000 ultrafast bootstraps. Support values correspond to multi-locus bootstraps and posterior probabilities, respectively.

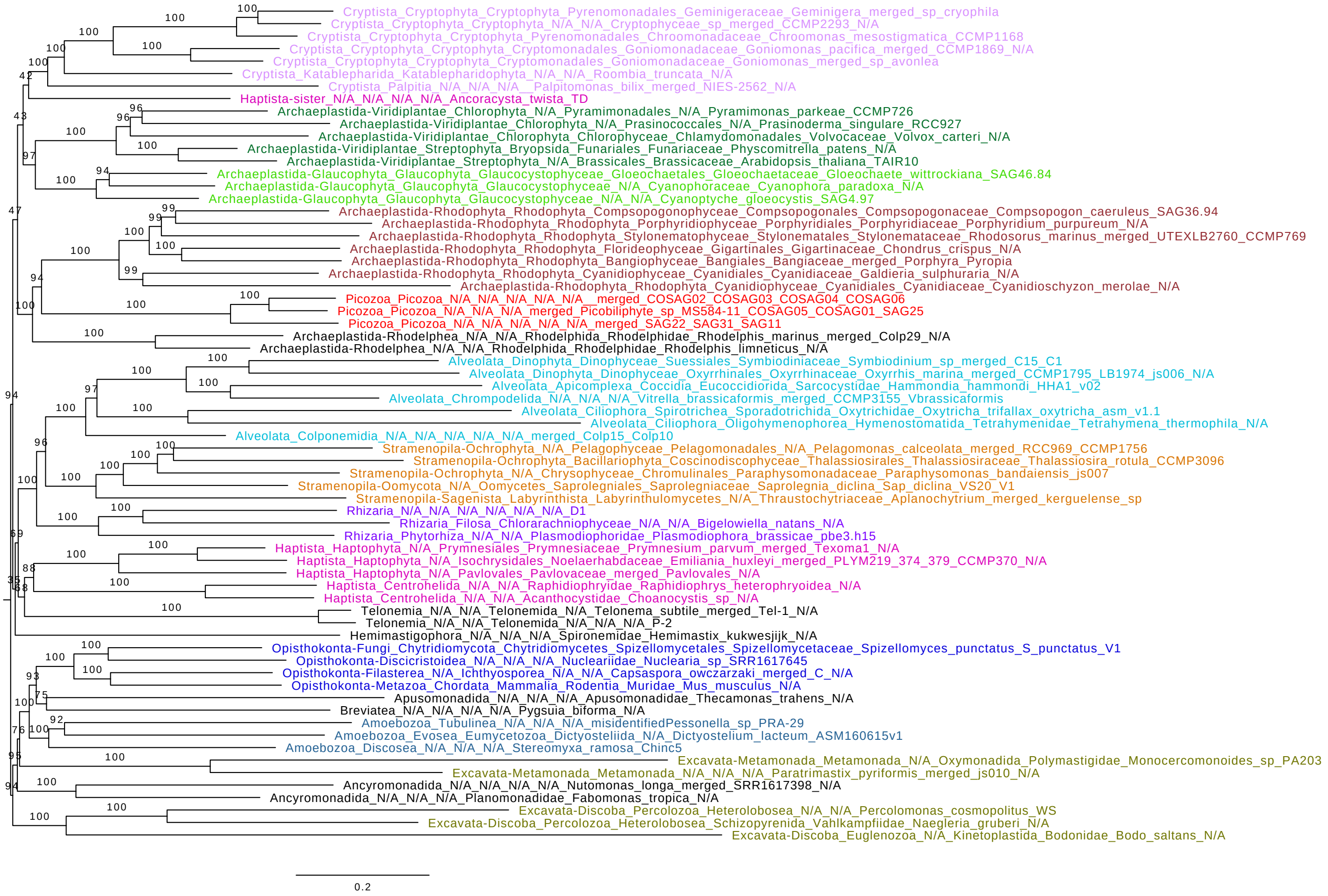

Supplementary Figure 7: Maximum likelihood tree of 67 eukaryotic species showing the position of Picozoa. The tree is based on the concatenated alignment of 317 marker genes, filtered with BMGE and trimmed of the 50% most heterogenous sites according to the chi-square statistic. The tree was reconstructed using the site-heterogeneous model LG+C60+F+G, support values correspond to 1000 ultrafast bootstrap replicates.

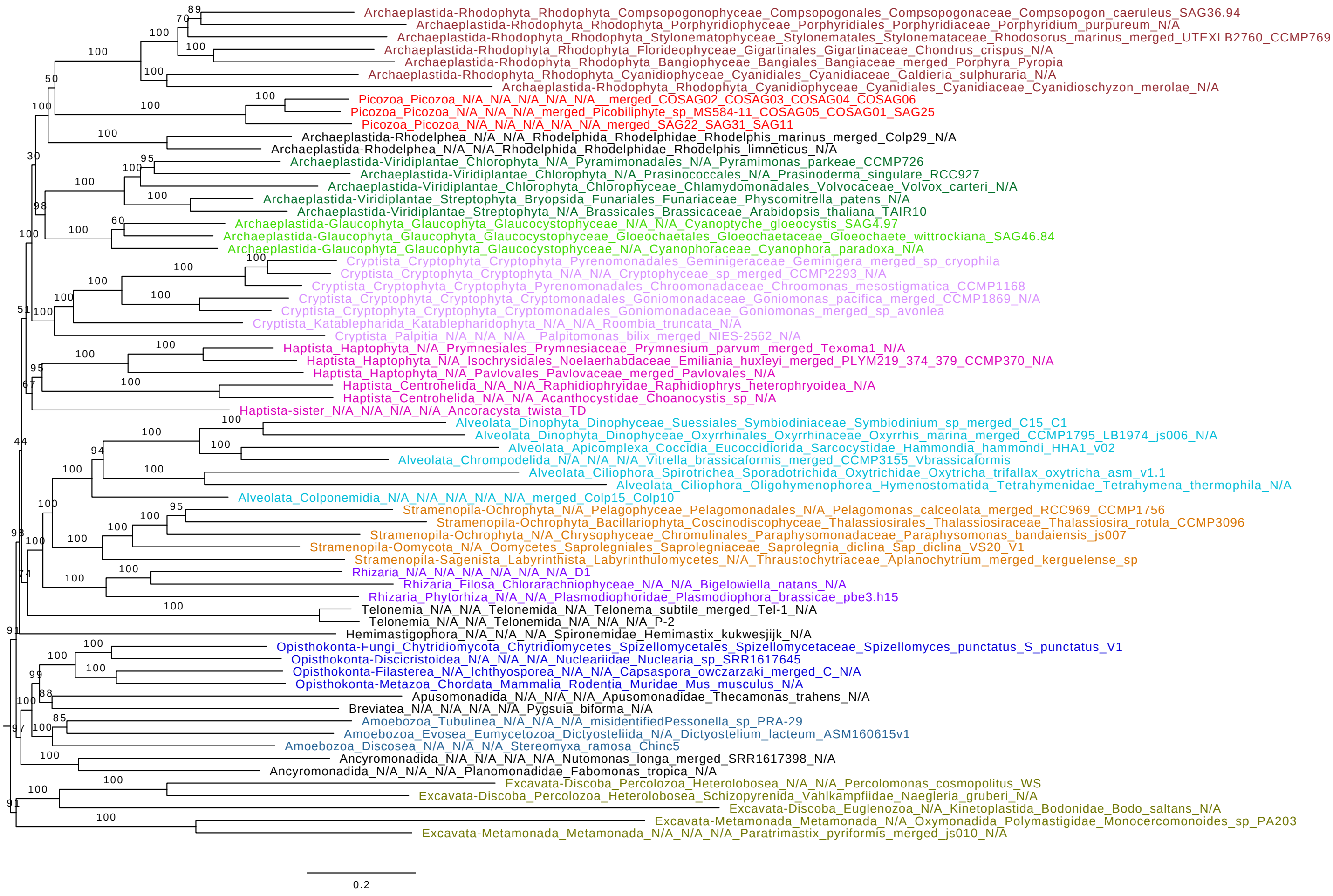

Supplementary Figure 8: Maximum likelihood tree of 67 eukaryotic species showing the position of Picozoa. The tree is based on the concatenated alignment of 224 marker genes with at least two monophyletic Picozoa sequences (filtered with BMGE). The tree was reconstructed using the site-heterogeneous model LG+C60+F+G, support values correspond to 1000 ultrafast bootstrap replicates.

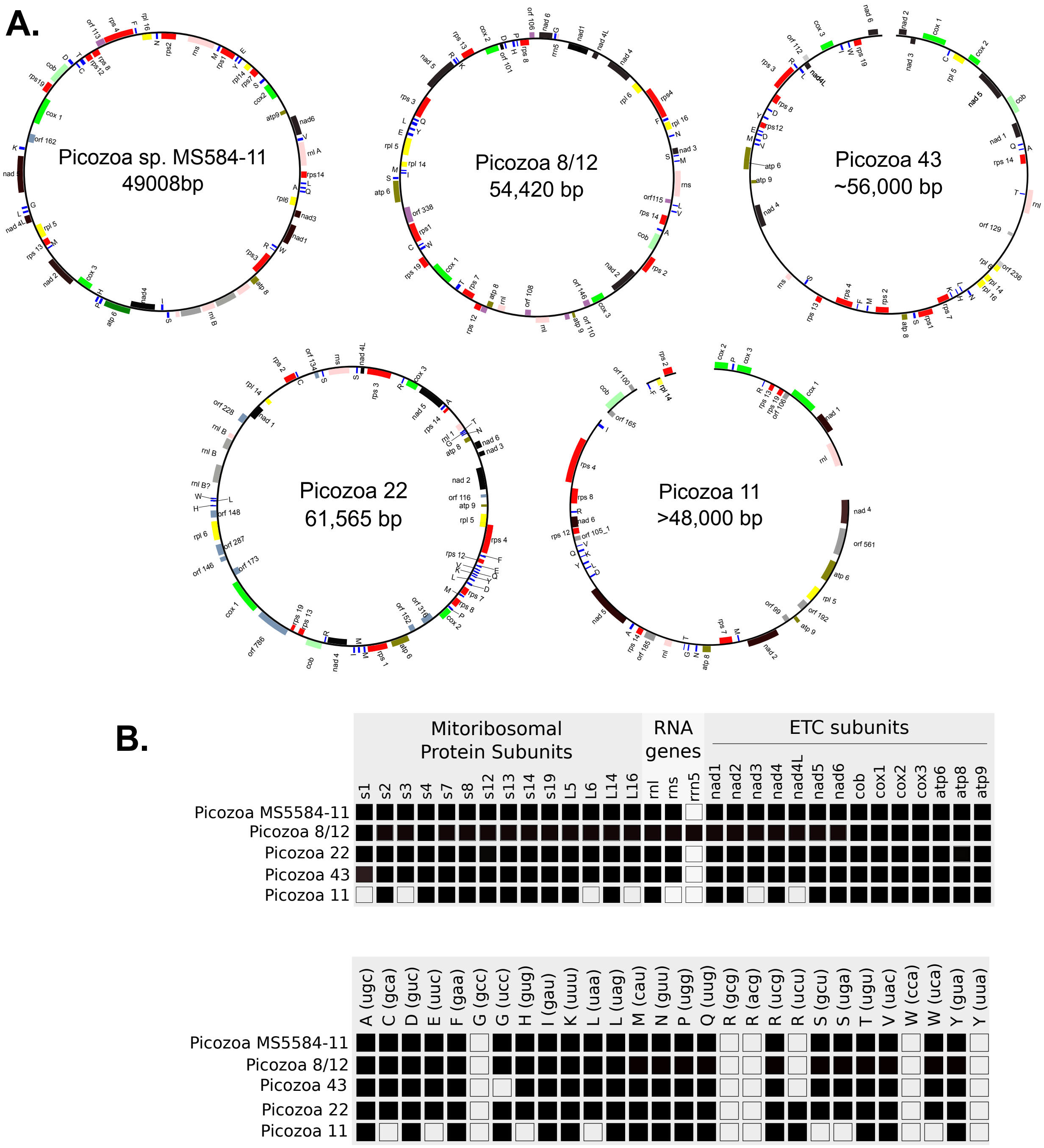

Supplementary Figure 9: Complete and near complete mitochondrial genomes assembled from diverse picozoan SAGs. A. Circular depictions of picozoan mitochondrial genomes compared to the published Picozoa MS584-11 sequence (Janouskovec et al. 2017). Mitochondrial contigs were annotated using mfannot with manual corrections as needed (http://megasun.bch.umontreal.ca/RNAweasel/). MtDNAs are represented as circular diagrams or broken circles if contigs could not be joined. For SAGs 8 and 12, the mitochondrial genome was inferred by stitching near-identical stretches together. Colour-coded genes: black, complex I; green, complex III; light green, complex IV; dark green, complex V; yellow, small ribosomal subunit proteins; red, large ribosomal subunit proteins; blue, tRNAs; pink, rnl and rns genes. B. gene complement of sequenced picozoan mitochondrial genomes. Top: protein coding and ribosomal RNA gene complement. Bottom: tRNA gene complement.

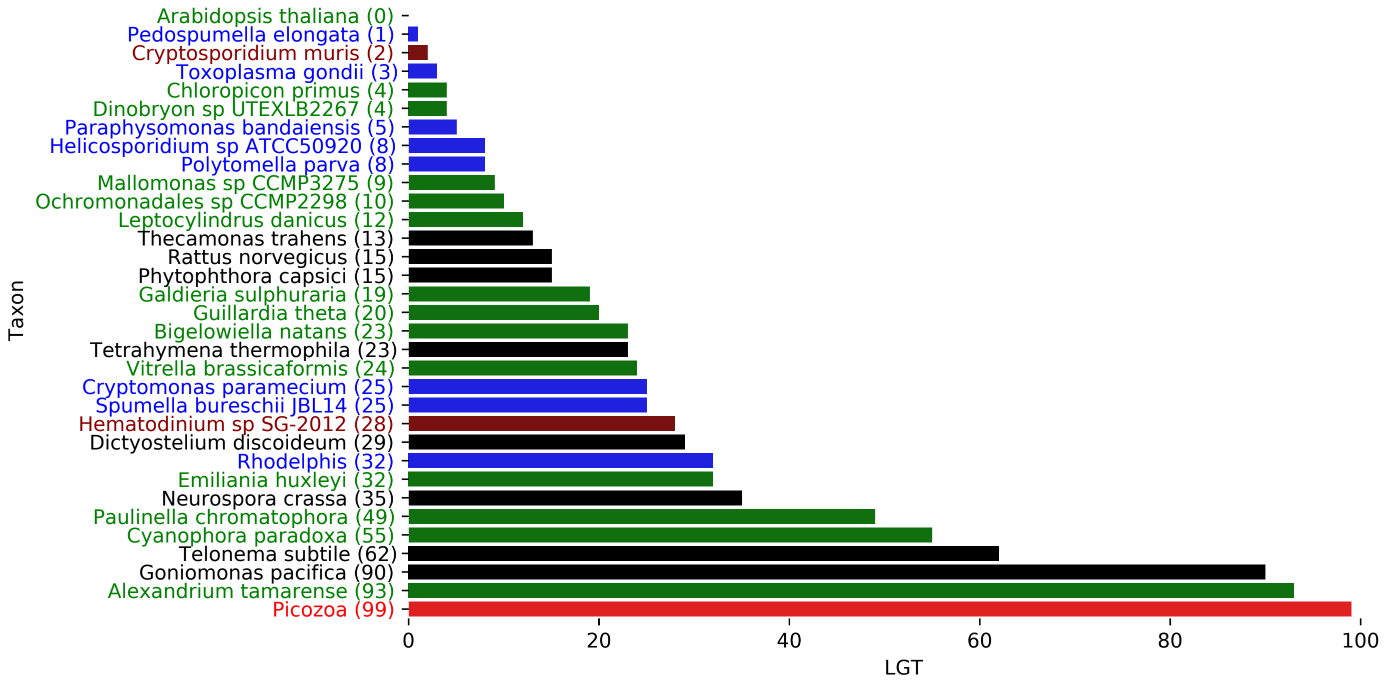

Supplementary Figure 10: Number of inferred lateral gene transfers (LGT) across a selection of 33 species. The species represent groups with a photosynthetic plastid (green), a non-photosynthetic plastid (blue), confirmed plastid loss (yellow) and no known plastid ancestry (black). These species serve as a comparison to Picozoa (orange).

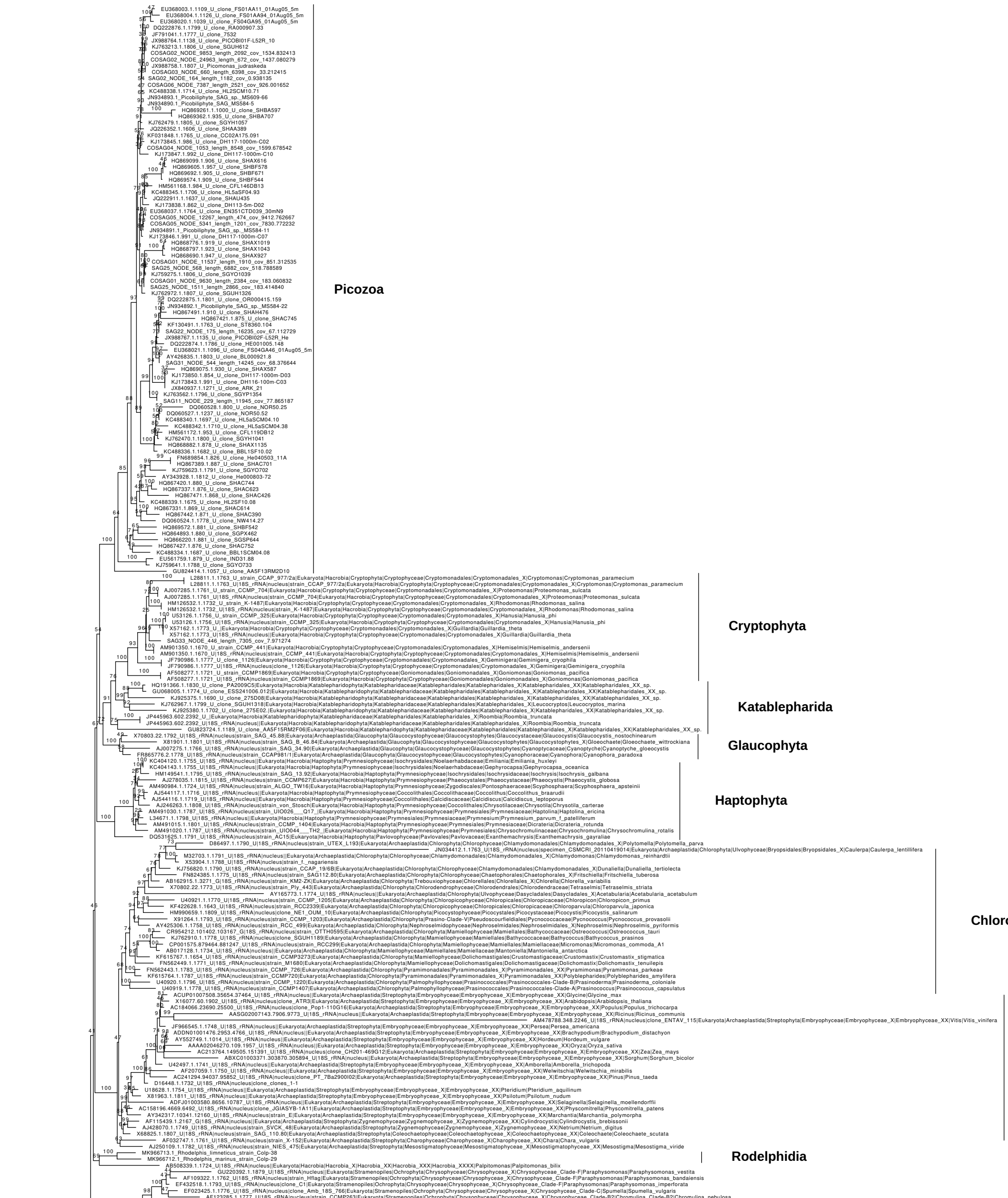

Supplementary Figure 11: Maximum Likelihood tree of the 18S rRNA gene from the individual SAG assemblies and an extended number of reference sequences from Picozoa and other major eukaryotic groups from the PR2 database. The tree was reconstructed under the model GTR+R5+F.

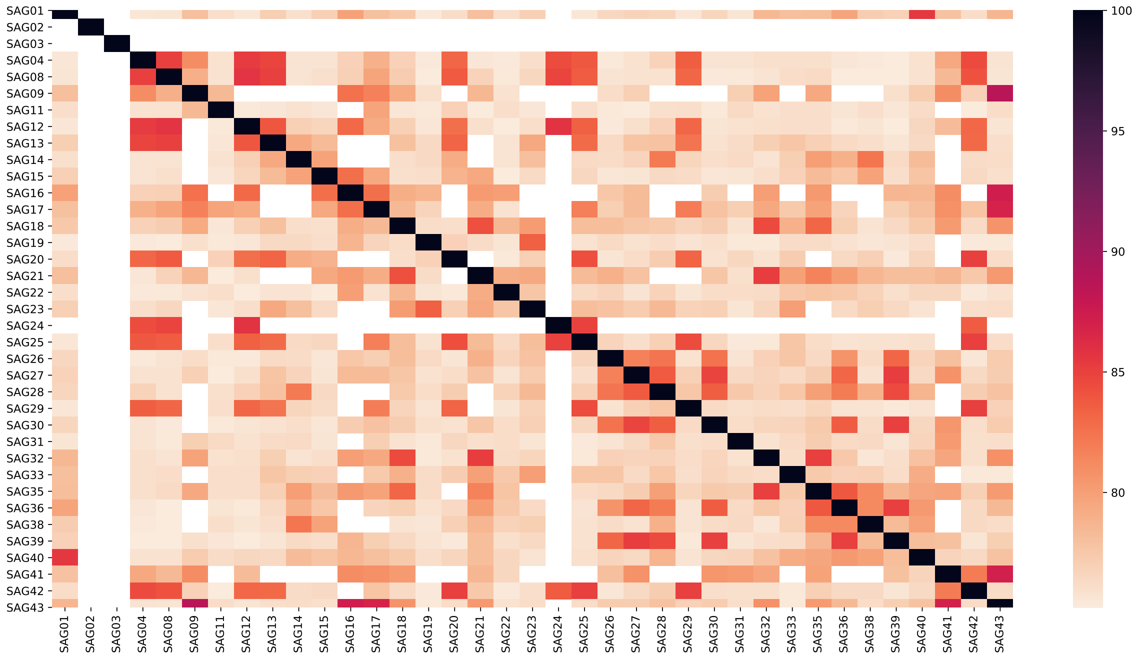

Supplementary Figure 12: Heatmap showing pairwise ANI for 43 initial picozoan SAGs as estimated with FastANI. Due to the incompleteness of the SAGs, many ANI values are zero, since there is no overlap between the assemblies.

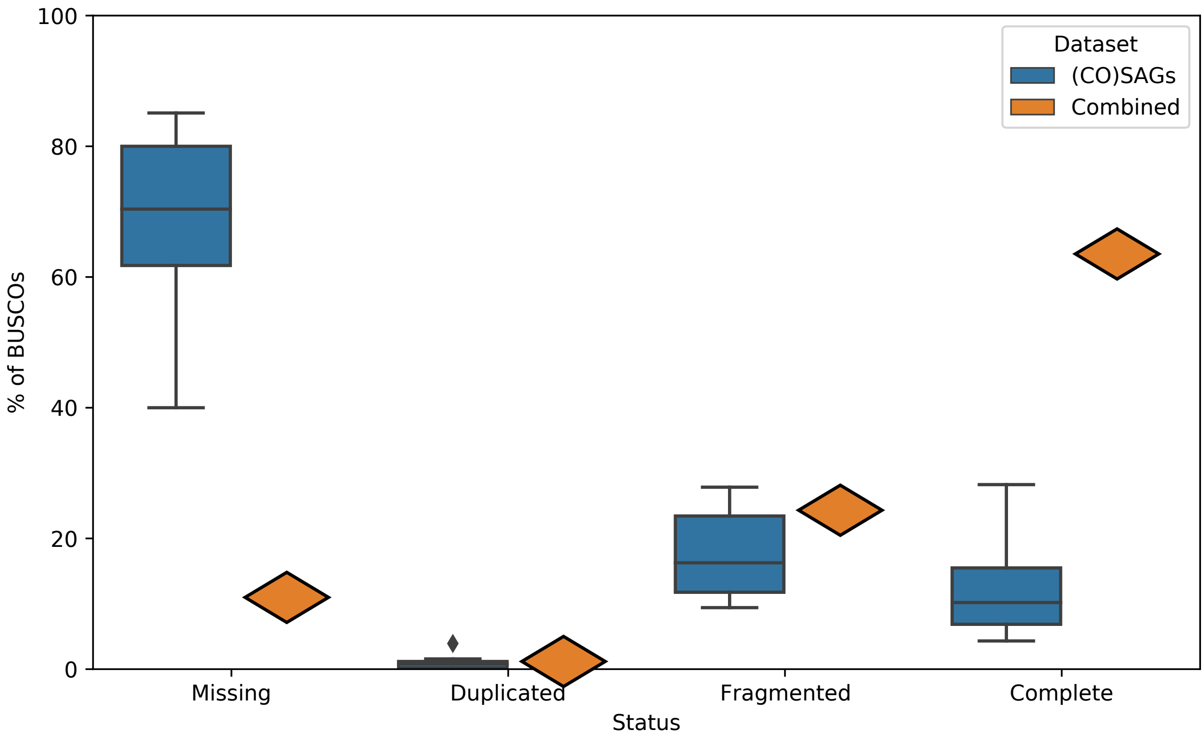

Supplementary Figure 13: Boxplots of different BUSCO categories (Missing, Complete, Fragmented and Duplicated) for all selected SAGs/Co-SAGs. Only SAGs that were used for Phylogenomic reconstruction were considered (n=10) as well as the results for all these 10 assemblies combined. For the combined value, a BUSCO was considered complete if it was complete in at least one assembly. The boxes show the minimum and maximum (excluding outliers), 1st quartile and 3rd quartile as well as the median. Source data are provided in a Source Data file.

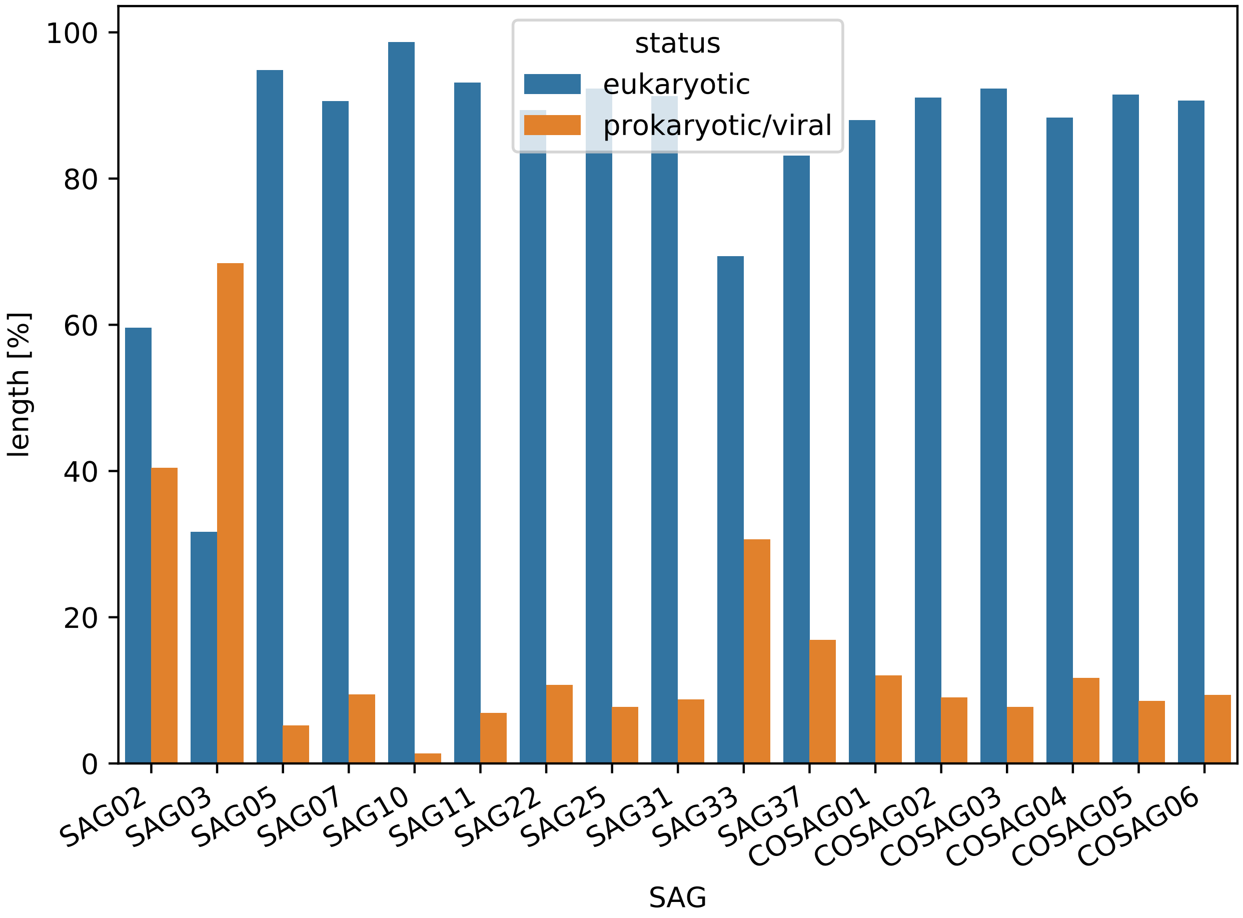

Supplementary Figure 14: Contamination estimate for each of the 17 final SAGs/Co-SAGs. All proteins were subjected to a DIAMOND blastp search against the ncbi NR database. If 60% of all proteins predicted from a contig only showed significant hits to prokaryotes or viruses, the contig was considered a putative contamination. Values correspond to fractions of contaminated/clean contigs of the total assembly length.

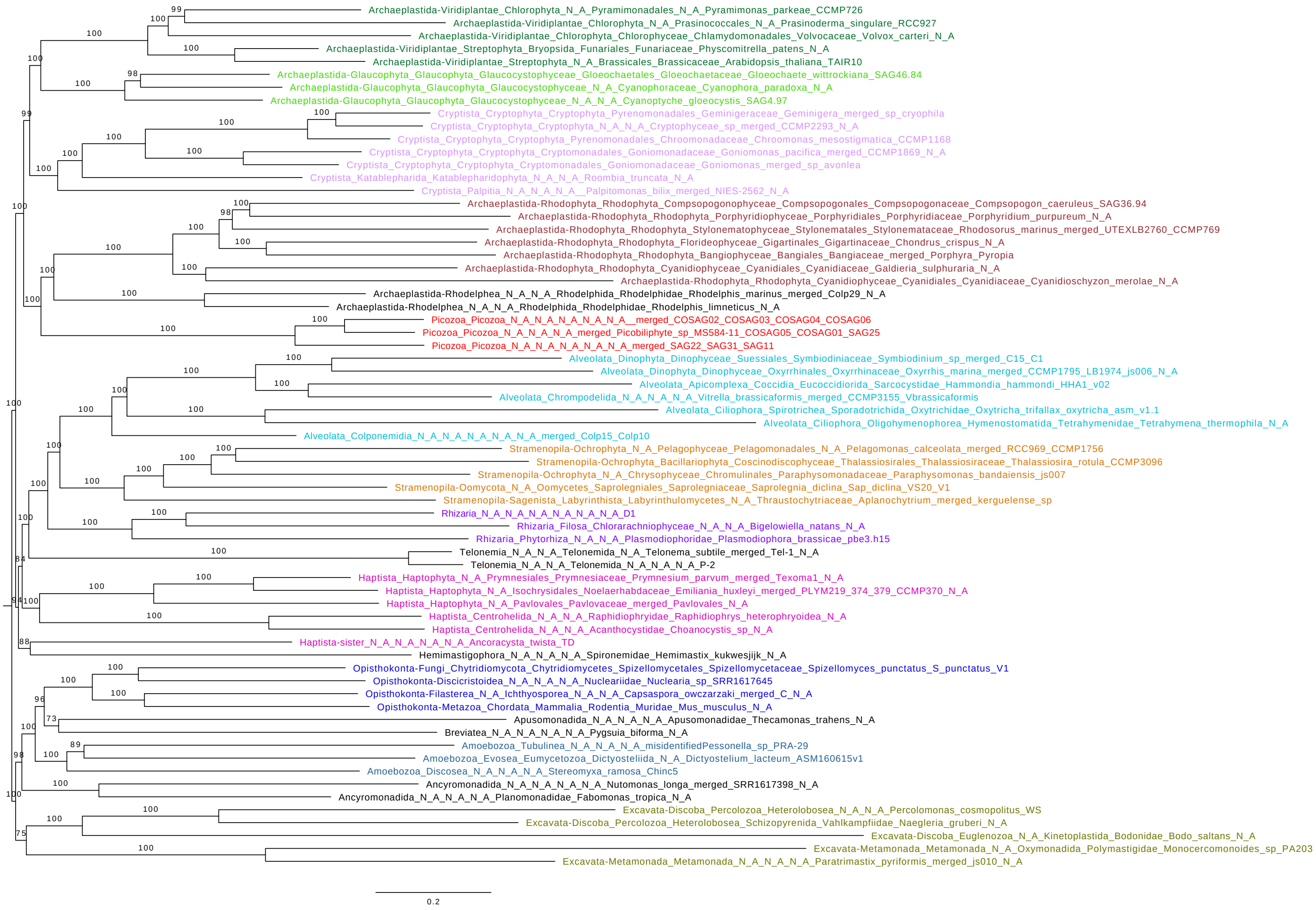

Supplementary Figure 15: Maximum likelihood tree of eukaryotic species showing the position of Picozoa. The tree is based on the concatenated alignment of 317 marker genes (filtered with Divvier) and was reconstructed using the PMSF approximation of the site-heterogeneous model LG+C60+F+G. Support values correspond to 100 non-parametric bootstrap replicates.

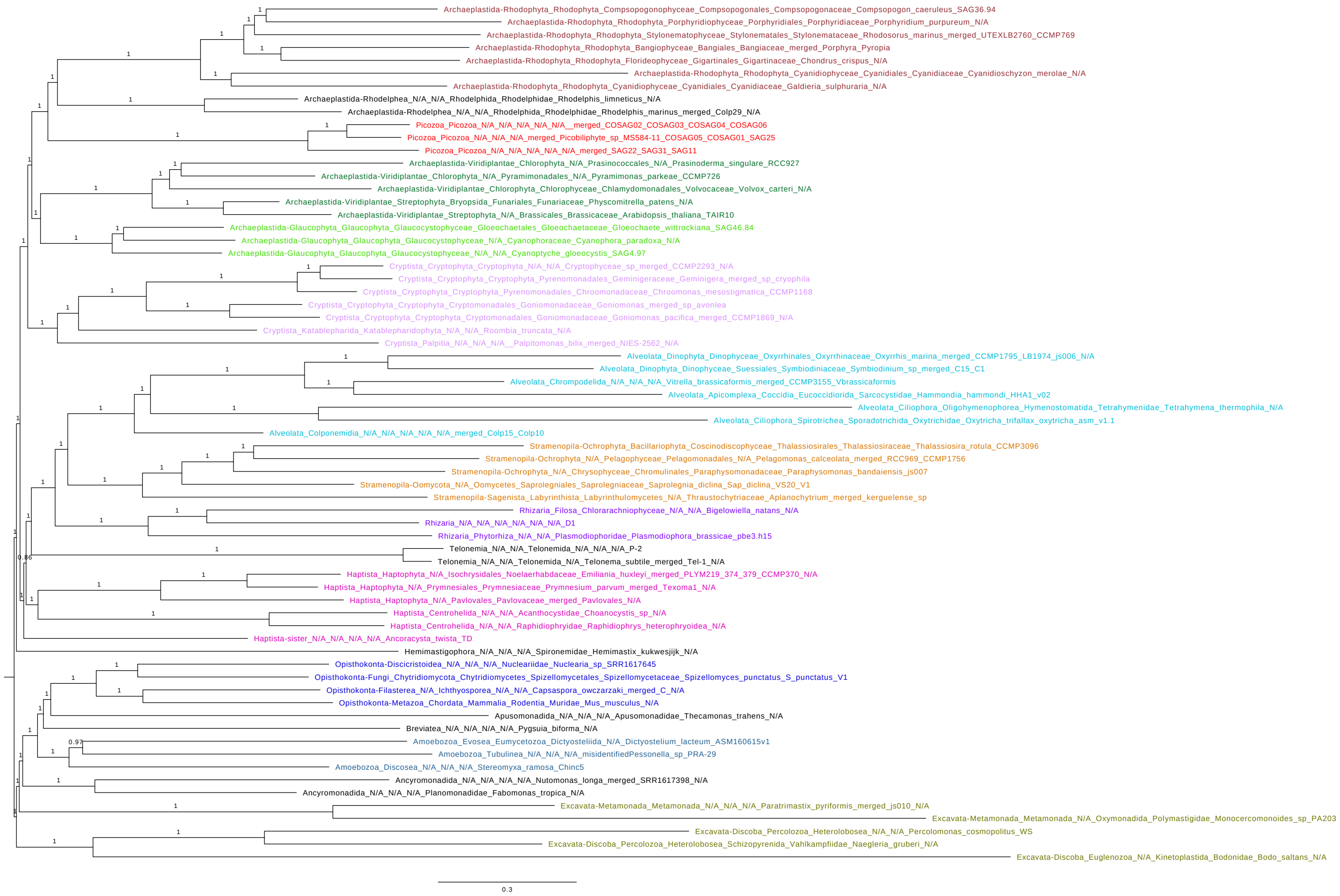

Supplementary Figure 16: Bayesian phylogenetic tree made using PhyloBayes. The tree is based on the concatenated alignment of 317 marker genes (filtered with BMGE). Shown is the consensus of chains 1 and 2 (maxdiff 0.26) after 3600 cycles with 1500 cycles removed as burn-in. Support values correspond to posterior probability values.

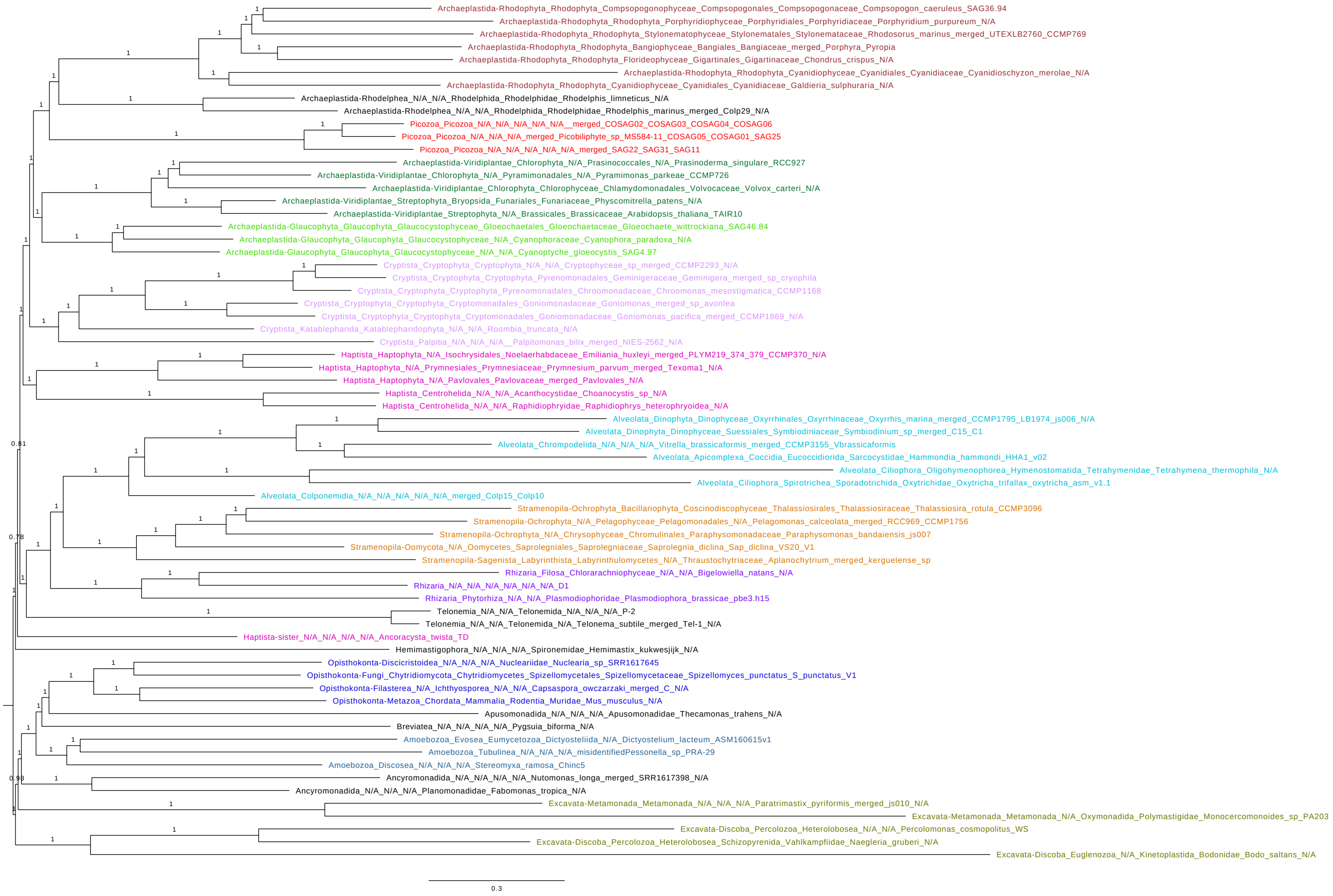

Supplementary Figure 17: Bayesian phylogenetic tree made using PhyloBayes. The tree is based on the concatenated alignment of 317 marker genes (filtered with BMGE). Shown is the consensus of chain 3 after 3600 cycles with 1500 cycles removed as burn-in. Support values correspond to posterior probability values.
