# Supplementary material for: Single cell genomics reveals plastid-lacking Picozoa are close relatives of red algae
Source: Nat Commun. 2021 Nov 17;12:6651. doi: 10.1038/s41467-021-26918-0 (PMC8599508; doi:10.1038/s41467-021-26918-0)
Supplement: Supplementary file 3 — Description of Additional Supplementary Files [file 41467_2021_26918_MOESM3_ESM.docx]

**Description of Additional Supplementary Files**

**Title: Supplementary Data 1**

Description: Assembly/genome characteristics SAGs and CO-SAGs

**Title: Supplementary Data 2**

Description: Phylogenomic dataset taxon selection and taxon merging

**Title: Supplementary Data 3**

Description: untrimmed EEF2 alignment showing 2 amino acid signature in Rhodelphis, Rhodophyte, Chloroplastida, Cryptista, Haptophytes (but neither centrohelids not Ancoracysta) and Picozoa

**Title: Supplementary Data 4**

Description: The Picozoan MS584-11 mitochondrial genome was used as a BLAST query into the 43 picozoan SAGs. Putative mitochondrial contigs were retrieved and used as queries into the NCBI nr database. If the top hit retrieved was MG202007.1, we considered the query sequence to be a bona fide mitochondrial contig.

**Title: Supplementary Data 5**

Description: Commonly retained plastid pathways and proteins

**Title: Supplementary Data 6**

Description: EGT clustering dataset taxon selection
