# Supplementary material for: Single cell genomics reveals plastid-lacking Picozoa are close relatives of red algae
Source: Nat Commun. 2021 Nov 17;12:6651. doi: 10.1038/s41467-021-26918-0 (PMC8599508; doi:10.1038/s41467-021-26918-0)
Supplement: Supplementary file 10 — Reporting Summary [file 41467_2021_26918_MOESM10_ESM.pdf]

nature research

Corresponding author(s): Fabien Burki

Last updated by author(s): Oct 13, 2021

Reporting Summary

Nature Research wishes to improve the reproducibility of the work that we publish. This form provides structure for consistency and transparency in reporting. For further information on Nature Research policies, see our Editorial Policies and the Editorial Policy Checklist.

Statistics

For all statistical analyses, confirm that the following items are present in the figure legend, table legend, main text, or Methods section.

n/a	Confirmed	
x		The exact sample size (*n*) for each experimental group/condition, given as a discrete number and unit of measurement
x		A statement on whether measurements were taken from distinct samples or whether the same sample was measured repeatedly
x		The statistical test(s) used AND whether they are one- or two-sided<br>*Only common tests should be described solely by name; describe more complex techniques in the Methods section.*
x		A description of all covariates tested
	x	A description of any assumptions or corrections, such as tests of normality and adjustment for multiple comparisons
x		A full description of the statistical parameters including central tendency (e.g. means) or other basic estimates (e.g. regression coefficient) AND variation (e.g. standard deviation) or associated estimates of uncertainty (e.g. confidence intervals)
x		For null hypothesis testing, the test statistic (e.g. *F*, *t*, *r*) with confidence intervals, effect sizes, degrees of freedom and *P* value noted<br>*Give P values as exact values whenever suitable.*
	x	For Bayesian analysis, information on the choice of priors and Markov chain Monte Carlo settings
x		For hierarchical and complex designs, identification of the appropriate level for tests and full reporting of outcomes
x		Estimates of effect sizes (e.g. Cohen's *d*, Pearson's *r*), indicating how they were calculated

Our web collection on statistics for biologists contains articles on many of the points above.

Software and code

Policy information about availability of computer code

Data collection	No Software was used for data collection
Data analysis	Trim Galore v0.6.1, SPAdes v3.13.0, Prodigal v2.6.3, MAFFT E-INS-i v7.429, fastANI v1.2, BUSCO v4.1.3, QUAST v5.0.2, HMMER v3.2.1, PREQUAL v1.02, Divvier v1.0, IQ-TREE v2.1.1, BMGE v1.12, Cutadapt v.1.135, DIAMOND v2.0.6, PhyloBayes MPI v1.8, GetOrganelle v1.7.1, trimAl v1.4.rev15, PhyloMagnet v0.7, OrthoFinder v2.4.0, VSEARCH v2.15.1, Sickle 1.33, Summit v 6.3.1, BD FACS Software v1.0.0.650. All custom scripts used in this study are available at https://github.com/maxemil/picozoa-scripts (doi:10.5281/zenodo.5561108) under a MIT license.

For manuscripts utilizing custom algorithms or software that are central to the research but not yet described in published literature, software must be made available to editors and reviewers. We strongly encourage code deposition in a community repository (e.g. GitHub). See the Nature Research guidelines for submitting code & software for further information.

Data

Policy information about availability of data

All manuscripts must include a data availability statement. This statement should provide the following information, where applicable:

- Accession codes, unique identifiers, or web links for publicly available datasets
- A list of figures that have associated raw data
- A description of any restrictions on data availability

All data used for the analyses as well as results files such as contigs and single gene trees are available at figshare (https://doi.org/10.6084/m9.figshare.c.5388176). A sequenceServer BLAST server was set up for the SAG assemblies: http://evocellbio.com/SAGdb/burki/. Raw sequencing reads were deposited in the Sequence Read Archive (SRA) at NCBI under accession PRJNA747736 (https://www.ncbi.nlm.nih.gov/bioproject/PRJNA747736). Public data that was used is available from pr2-database.org/, and from the accesssions and weblinks provided in Supplementary Table 1 and Supplementary Data 6.

Field-specific reporting

Please select the one below that is the best fit for your research. If you are not sure, read the appropriate sections before making your selection.

☐ Life sciences ☐ Behavioural & social sciences ☒ Ecological, evolutionary & environmental sciences

For a reference copy of the document with all sections, see nature.com/documents/nr-reporting-summary-flat.pdf

Ecological, evolutionary & environmental sciences study design

All studies must disclose on these points even when the disclosure is negative.

Study description	Phylogenomic analysis of eukaryotes, with focus on Picozoa
Research sample	Genomic data generated for 43 cells of Picozoa and publicly available genomes from a collection of eukaryotic and bacterial species. source of the public data can be obtained from Supplementary Table 1 and Supplementary Data 6. The 43 SAGs represent all previously delineated groups within Picozoa.
Sampling strategy	Cells corresponding to Picozoa were sorted from natural population. Publicly available data were chosen to span a wide diversity of eukaryotic lineages and show high genome/transcriptome completeness (i.e., high number of protein-coding genes used for phylogenetic analyses). Sampling size not applicable.
Data collection	Data were downloaded from public databases, or generated as part of this study.
Timing and spatial scale	Baltic sea: Surface (depth: up to 2 m) marine water was collected from the Linnaeus microbial Observatory (LMO) in the Baltic Sea located at 56°N 55.85’ and 17°E 03.64’ on two occasions: 2 May 2018 (6.1°C and 6.8 ppt salinity) and 3 April 2018 (2.4°C and 6.7 ppt salinity).<br>Eastern North Pacific: The stations where sorting occurred were located at 36.748°N, 122.013°W (Station M1; 20 m, 2 April 2014 and 10 m, 5 May 2014); 36.695°N, 122.357°W (Station M2, 10 m, 5 May 2014); and 36.126°N, 123.49°W (Station 67-70, 20 m 15 October 2013).
Data exclusions	While building the phylogenomic dataset, sequences likely representing contaminants or paralogs were removed upon gene tree inference and visualisation
Reproducibility	All procedures are accurately described in the Methods, with software versions and parameterizations. The initial and curated Datasets are provided on FigShare. In general results were only accepted is confirmed by several SAGs, thus ensuring reproducibility.
Randomization	Bootstrap replicates for Phylogenetic analysis were generated in a randomized manner.
Blinding	No blinding was applied as no statistical tests were performed where blinding could be applied.

Did the study involve field work? ☒ Yes ☐ No

Field work, collection and transport

Field conditions	Surface oceanic waters, temperature between 2-6 degrees C.<br>Sampling dates:<br>Baltic sea: 2 May 2018 and 3 April 2018<br>Eastern North Pacific: 2 April 2014, 5 May 2014, 15 October 2013
Location	Baltic sea: Surface (depth: up to 2 m) marine water was collected from the Linnaeus microbial Observatory (LMO) in the Baltic Sea located at 56°N 55.85’ and 17°E 03.64’.<br>Eastern North Pacific: The stations where sorting occurred were located at 36.748°N, 122.013°W (Station M1; 20 m, and 10 m); 36.695°N, 122.357°W (Station M2, 10 m); and 36.126°N, 123.49°W (Station 67-70, 20 m).
Access & import/export	No permit required
Disturbance	No disturbance was caused, only sampled a small amount of surface oceanic waters

Reporting for specific materials, systems and methods

We require information from authors about some types of materials, experimental systems and methods used in many studies. Here, indicate whether each material, system or method listed is relevant to your study. If you are not sure if a list item applies to your research, read the appropriate section before selecting a response.

Materials & experimental systems

n/a	Involved in the study
☒	☐ Antibodies
☒	☐ Eukaryotic cell lines
☒	☐ Palaeontology and archaeology
☒	☐ Animals and other organisms
☒	☐ Human research participants
☒	☐ Clinical data
☒	☐ Dual use research of concern

Methods

n/a	Involved in the study
☒	☐ ChIP-seq
☐	☒ Flow cytometry
☒	☐ MRI-based neuroimaging

Flow Cytometry

Plots

Confirm that:

☐ The axis labels state the marker and fluorochrome used (e.g. CD4-FITC).

☐ The axis scales are clearly visible. Include numbers along axes only for bottom left plot of group (a 'group' is an analysis of identical markers).

☐ All plots are contour plots with outliers or pseudocolor plots.

☐ A numerical value for number of cells or percentage (with statistics) is provided.

Methodology

Sample preparation	Oceanic waters
Instrument	MoFlo Astrios EQ cell sorter (Beckman Coulter)<br>BD InFlux Fluorescently Activated Cell Sorter
Software	Summit v 6.3.1<br>BD FACS Software v1.0.0.650
Cell population abundance	Abundance was not determined, but the presence of target cells was determined by PCR using specific probes
Gating strategy	For MoFlo:<br>Gates were set mainly based on Mitotracker intensity and the dye was detected by a 488 nm and 640 nm laser for excitation, 100 µm nozzle, sheath pressure of 25 psi and 0.1 µm sterile filtered 1 x PBS as sheath fluid.<br><br>For BD inFlux:<br>Two different stains were used: LysoSensor and LysoTracker, or both together. Selection of eukaryotic cells stained with LysoTracker Green DND-26 (Life Technologies; final concentration, 25 nM) was based on scatter parameters, positive green fluorescence (520/35 nm bandpass), as compared to unstained samples, and exclusion of known phytoplankton populations, as discriminated by their forward angle light scatter and red (chlorophyll-derived) autofluorescence (i.e., 692/40 nm bandpass) under 488 nm excitation, similar to methods in49. Likewise, selection of cells stained with LysoSensor Blue DND-167 (Life Technologies; final concentration, 1 µM), a ratiometric probe sensitive to intracellular pH levels, e.g. in lysosomes, was based on scatter parameters, positive blue fluorescence (435/40 nm bandpass), as compared to unstained samples, and exclusion of known phytoplankton populations, as discriminated by their forward angle light scatter and red (chlorophyll-derived) autofluorescence (i.e., 692/40 nm bandpass filter) under 355 nm excitation. For sorts using both stains all of the above criteria, and excitation with both lasers (with emissions collected through different pinholes and filter sets), were applied to select cells

☐ Tick this box to confirm that a figure exemplifying the gating strategy is provided in the Supplementary Information.
