# Supplementary material for: Single cell genomics reveals plastid-lacking Picozoa are close relatives of red algae
Source: Nat Commun. 2021 Nov 17;12:6651. doi: 10.1038/s41467-021-26918-0 (PMC8599508; doi:10.1038/s41467-021-26918-0)
Supplement: Supplementary file 2 — Peer Review File [file 41467_2021_26918_MOESM2_ESM.pdf]

nature portfolio

Peer Review File

Single cell genomics reveals plastid-lacking Picozoa are close relatives of red algae

Open Access This file is licensed under a Creative Commons Attribution 4.0 International License, which permits use, sharing, adaptation, distribution and reproduction in any medium or format, as long as you give appropriate credit to the original author(s) and the source, provide a link to the Creative Commons license, and indicate if changes were made. In the cases where the authors are anonymous, such as is the case for the reports of anonymous peer reviewers, author attribution should be to 'Anonymous Referee' followed by a clear attribution to the source work. The images or other third party material in this file are included in the article's Creative Commons license, unless indicated otherwise in a credit line to the material. If material is not included in the article's Creative Commons license and your intended use is not permitted by statutory regulation or exceeds the permitted use, you will need to obtain permission directly from the copyright holder. To view a copy of this license, visit http://creativecommons.org/licenses/by/4.0/.

Reviewers' Comments:

Reviewer #1:
Remarks to the Author:
Using the single-cell genomics from natural picozoan samples, in conjunction with phylogenomics to infer their evolutionary history, the authors suggest that Picozoa is a plastid-lacking Archaeplastida lineage that is sister to Rhodophyta in the eukaryotic tree. This is a very interesting paper, however, two critical issues must be addressed; i) validation of SAG data with additional analysis, ii) consideration of biological traits (e.g., common genes, morpho-physiological evidence) of Picozoa and Rhodophyta. Aside from the phylogeny, I don't see any other evidence to support the common ancestry of Picozoan lineages with red algae as well as with other Archaeplastida lineages. Moreover, the phylogenies used by the authors have conflicts among them, therefore, the results are unconvincing. The author's main idea of Picozoa being a sister to Rhodophyta is a surprising result for Archaeplastida evolution in general, and has the potential to revise the paradigm of primary endosymbiosis. Therefore, without additional lines of evidence, I cannot recommend this manuscript for publication.

DETAILED COMMENTS:
Data validation and gene modeling issue
Misidentification of picozoan genes could lead to critical analytical flaws, which may lead to wrong interpretations, therefore, the results need more careful validation. Because the SAG data generates viral, bacterial, or other eukaryotic genome sequences from undigested DNA fragments in the single cell, which has been shown in a previous study (Yoon et al., 2011), the authors need to provide more careful validation and information about the portion of the picozoan genome in the total assembled genome data in a table or figure. Indeed, the authors excluded SAG33 because of significant cryptophyte sequence contamination. It has been previously been demonstrated that the Picozoa - cryptophyte connection (maybe other small eukaryotes as well) can represent fragments of undigested genomes in the cell. Any exogenous genes from contaminants could lead the misplacement of Picozoa, therefore, robust validation is needed, especially for all the individual gene sets (317 gene markers). I expect to see a list of individual genes that support the monophyly of Picozoa, Rhodelphida, and red algae, even if single gene contains less information to resolve this relationship. If the multiple picozoan taxa did not group together, this gene should be considered to exclude from the concatenated dataset (because it is likely contaminated from different eukaryotes).
For another example, I was able to detect a Achromobacter 16S rDNA sequence with 100% identity and complete query coverage from 'NODE_30_length_5267_cov_9249.629063 (position: 2,413 to 887)' in the SAG02 assembly. Again, what if the authors included this kind of bacterial genes in the phylogenetic analysis, it would mislead the phylogeny. BlobTools (Laetsch & Blaxter, 2017) or Kraken2 (Wood et al., 2019) can be used to sort out viral or bacterial contaminations. BUSCO scores may be overestimated in this study due to some bacterial genes that are homologous to eukaryotic genes. BUSCO and CheckM (Parks et al., 2015) would be used for assessing the genome completeness of Picozoa. The authors simply combined partial and complete BUSCO genes, which I assume are mostly partial, to represent BUSCO in the manuscript. Furthermore, I was not able to find information about duplicated BUSCO genes, which would be required to assess their SAG genome accurately. Recovering complete picozoan genes is difficult due to methodological limitations of WGA, but readers should be made aware of this limitation. More importantly, the authors predicted Picozoa genes using prodigal, a prokaryotic genome prediction program, however, eukaryotic gene prediction needs to be done by considering intron splicing patterns. Because we don't know anything about introns in picozoan species yet, gene prediction should be done using tools that are typically used for eukaryotic gene modeling. BUSCO (with '-m geno' option) and AUGUSTUS (Stanke et al., 2006) with the training sets would be used to obtain gene structure information. Furthermore, genome correction (e.g., Pilon) using Illumina raw data will reduce gene modeling errors by refining the genome assembly.

Phylogenetic analysis
Although, the Picozoa have shown a strong monophyly with reds or Rhodelphis lineages, the strong monophyly supported in Archaeplastida or other lineages does not fit with conventional views, for example, the monophyly of Crytophyta and Rhodophyta (Fig. S3). Even though previous phylogenetic trees (Moreira & López-Garca, 2014) haven't showed high support values of picozoan

lineage, an approximately unbiased (AU) test should be performed with all possible phylogenetic positions (especially to the topology of Archaeplastida without Picozoa). Therefore, I feel that those phylogenies were chosen by the author as a form of "cherry picking", because of their inconsistency with previous studies (Burki et al., 2012) and even the current study (Fig. S3). Because these results have the potential to alter our understanding of Archaeplastida evolution, I would suggest approaching this issue with a more conservative aspect. Contamination candidates or significantly divergent sequences that could mislead the phylogenetic signal should be deleted from the dataset. For example, using BMGE alignments from authors' dataset (from figshare 'Picozoa: 67 taxa alignments and trees'; 67 taxa, 317 genes), Fabomonas tropica in xpb phylogeny and Rhodelphis limneticus in BMS phylogeny (see figures below) showed relatively long branches compared to other eukaryotic lineages from gene trees reconstructed by IQ-TREE (-m TEST; 1K ultrafast bootstraps).

Additionally, I reconstructed the phylogenetic trees (from figshare 'Picozoa: cleaned single genes, initial supermatrix tree'; 760 taxa, 317 genes, midpoint rooting), and I was still able to find some paraphyletic signal, which might imply the contaminations or paralogs in their dataset (e.g., red algae paralogs marked with red color in the phylogeny of nhp2 as follows).

Furthermore, as mentioned earlier, picozoan genes used for this study should support monophyly of at least two picozoan species in a single gene tree. I found that 52 genes out of 317 have a single taxon of picozoa from the final dataset used in the main figure. To test whether these datasets support the current phylogenetic relationship, I would like to see, 1) a new phylogeny based on combined genes only with multiple picozoan taxa, and 2) combined gene set only with picozoan taxa (exclude genes without picozoan taxa). These reduced and conserved datasets would provide more accurate evolutionary history.
In page 8, "This search resulted in one protein (Arogenate dehydrogenase) with picozoan homologues that were closely related to red algae and belonged to a larger clade with host-derived plastid targeted plant sequences, but neither the picozoan nor the red algal sequences displayed predicted transit peptides.". Provide phylogenetic tree regarding this gene.
I'm still skeptical of using concatenated data without serious validation step. Many phylogenetic studies have proven that just combing datasets does not always give a clear answer for species tree. For example, in this study, same dataset but different taxon sampling (794 taxa in Fig. S3) provided completely different phylogenetic relationship, especially Picozoa + Rhodelphia + Cryptopyta + Reds with strong bootstrap support (100%). To reach a correct species tree, I would suggest conducting other phylogenetic approaches (e.g., coalescence analysis - ASTRAL-III [Zhang et al., 2018], concordance tree - BUCKy [Ané et al., 2007]) as mentioned in these studies (Lemmon and Lemmon, 2013; Simmons & Gatesy, 2015). In addition, as found in recent study (Irisarri et al., 2021), very careful data and phylogeny validation were conducted (e.g., random sample of amino acid positions, quantifying gene support, normalized Robinson-Foulds distance, novel alignment descriptive metrics), which I was expecting in this current study.
Along with these suggestions, I am curious about the phylogeny using mitochondrial genome data, because the authors completed (or nearly completed) five mitochondrial genomes. Janouškovec, J. et al. (2017) already provided mitochondrial genome phylogeny with relatively strong bootstrap support values among major eukaryotic groups. If the authors provide a phylogeny using mitochondrial genomes data, then it will be one good evidence to support their hypothesis.
In a same line, phylogeny independent evidences such as amino acid insertion/deletion (e.g., EEF2 gene - Kim and Graham, 2008), list of sharing gene comparison between Picozoa, red algal and/or Archaeplastida, and morpho-physiological evidences (e.g., TEM) from Picomonas judraskeda (Seenivasan et al. 2013). Lacking both flagella and centriole are unique red algal characters, and these related genes would be good candidate to present.
Overall, limited taxon sampling, contamination or paralogs issues (which need to be addressed manually), and well-supported conflicts among trees (e.g., Fig. 2 vs. Fig. S3) continue to be major concerns that weaken the results presented in this paper and bring into question its major conclusion.

Reviewer #2:
Remarks to the Author:

This article presents the analysis of 43 single-cell genomes of the poorly-known heterotrophic protist group Picozoa. Phylogenetic analyses based on a large concatenation of conserved proteins support that Picozoa branch within the Archaeplastida, as sister group to red algae and the phagotrophic rhodelphids. Nevertheless, despite this phylogenetic position, the authors did not find any clear genomic evidence for the present or past presence of plastids in these organisms. These are unexpected results that suggest that Picozoa may be the first known case of a completely plastid-lacking lineage within the Archaeplastida.

I think that these are very interesting results and I only have relatively minor comments.

- Line 97. It would be useful that the authors give a short explanation of what is "Pico-PCR".

- Line 98: "The sequencing reads were assembled into genomic contigs, ranging in length from 350 kbp to 66 Mbp". Does it mean that all contigs were >350 kbp, up to 66 Mbp? Or do you refer to the total sum of contig sizes? It is unclear.

- Line 115: "The deep-branching picozoan lineages identified by Moreira and López-García (2014), as well as other possibly early-diverging lineages were not represented in our data". The delimitation of lineages is always arbitrary but I think that the groups 'BP2' and 'Deep branching 1' could be considered to form a single group (including also SAG11). In that case, some SAGs would be 'Deep branching 1' representatives.

- Line 168: "An approximately unbiased (AU) test rejected the topology where Picozoa branched with rhodelphids (p=0.0362), but the position of Picozoa as the closest sister to red algae could not be rejected (p=0.236)". Did the authors do a similar test with Picozoa as sister group to Archaeplastida? I think it is an important possibility that has to be tested too. Testing topologies where Cryptista branch within Archaeplastida (as it has been reported in previous articles) can also be very interesting with this dataset enriched in picozoan taxa.

- In relation with the latter point, this region of the phylogeny of eukaryotes has been very unstable in recent years and here the authors present a tree that is, once again, very different from previous ones. Therefore, it is important to be cautious and provide as much evidence as possible. For example, it would be very interesting to show the result of a slow-fast analysis to see if fast evolving sites influence this tree topology.

- Line 252: "This position has important implications for our understanding of plastid origins because, in contrast to all other archaeplastids known to date, our results strongly indicate that Picozoa lack plastids and plastid-associated EGTs". I think this sentence is too strong, the authors found some candidates and, despite the EGT/HGT ratio, we cannot completely exclude that they represent real EGT cases.

- Line 267: "A related explanation could involve a secondary endosymbiosis where the plastid in red algae, for example, was secondarily acquired from a green alga. The latter scenario would be effectively refuted by the identification of host-derived plastid components shared between all archaeplastid lineages". If the hosts are closely related, can you imagine to observe "shared" host-derived plastid components derived from convergent acquisitions?.

REVIEWER COMMENTS

We reproduced below the reviewers' remarks verbatim, providing a point-by-point response to all comments/concerns (in blue). We have extensively revised our manuscript to address the comments by the reviewers, please see the details below.

Reviewer #1 (Remarks to the Author):

Using the single-cell genomics from natural picozoan samples, in conjunction with phylogenomics to infer their evolutionary history, the authors suggest that Picozoa is a plastid-lacking Archaeplastida lineage that is sister to Rhodophyta in the eukaryotic tree. This is a very interesting paper, however, two critical issues must be addressed; i) validation of SAG data with additional analysis, ii) consideration of biological traits (e.g., common genes, morpho-physiological evidence) of Picozoa and Rhodophyta. Aside from the phylogeny, I don't see any other evidence to support the common ancestry of Picozoan lineages with red algae as well as with other Archaeplastida lineages. Moreover, the phylogenies used by the authors have conflicts among them, therefore, the results are unconvincing. The author's main idea of Picozoa being a sister to Rhodophyta is a surprising result for Archaeplastida evolution in general, and has the potential to revise the paradigm of primary endosymbiosis. Therefore, without additional lines of evidence, I cannot recommend this manuscript for publication.
We would like to thank the reviewer for the general interest in our work, and express our full gratitude for the highly detailed comments and in-depth review of our manuscript. The provided suggestions have greatly improved our analyses which now further strengthen the paper's main original conclusions.

DETAILED COMMENTS:
Data validation and gene modeling issue
Misidentification of picozoan genes could lead to critical analytical flaws, which may lead to wrong interpretations, therefore, the results need more careful validation. Because the SAG data generates viral, bacterial, or other eukaryotic genome sequences from undigested DNA fragments in the single cell, which has been shown in a previous study (Yoon et al., 2011), the authors need to provide more careful validation and information about the portion of the picozoan genome in the total assembled genome data in a table or figure.
We fully agree with the reviewer that SAGs from environmental samples are rarely truly single cells as associated cells (often unwanted) are jointly amplified, as is undigested foreign DNA. This was well demonstrated in the mentioned pioneering work on eukaryotic SAGs (Yoon et al. 2011), and has been repeatedly shown ever since. Determining the bacterial (or archaeal) or viral component in our data is relatively straightforward based on the large evolutionary distance that separates these from Picozoa, however the same is not true for possible eukaryotic contamination. In the case of eukaryotes, it is extremely difficult to automatically predict with confidence the level of foreign eukaryotic sequences over the entire assemblies for species like Picozoa with poor genome representation and no close relatives with high-quality genomes (or transcriptomes). To better assess and present the level of prokaryotic/viral contamination, we have now performed DIAMOND blast searches of the predicted proteins from the picozoa contigs against the nr database, and deemed contaminant those contigs with at least 60% of the encoded proteins showing only hits to prokaryotes or viruses. We found that up to around 10% of the SAGs/COSAGs used for phylogenomic analyses were contaminants according to this definition. We describe this new analysis in the method section (on p.12, last paragraph before the section *phylogenomics*) and present it in a new supplementary figure (S14). As an additional important check (and more specifically for detecting eukaryotic contamination), we manually evaluated all single-gene trees used to build the phylogenomic dataset for sequences that could derive from organisms other than Picozoa; when detected, the corresponding sequences were removed. Importantly, we found that in a majority of cases (252), sequences from more than one SAG grouped together and were not placed in suspicious positions in the trees (i.e. close to known groups), strongly suggesting that these were not contamination.

Indeed, the authors excluded SAG33 because of significant cryptophyte sequence contamination. It has been previously been demonstrated that the Picozoa - cryptophyte connection (maybe other small eukaryotes as well) can represent fragments of undigested genomes in the cell. Any exogenous genes from contaminants could lead the misplacement of Picozoa, therefore, robust validation is needed, especially for all the individual gene sets (317 gene markers). I expect to see a list of individual genes that support the monophyly of Picozoa, Rhodelphida, and red algae, even if single gene contains less information to resolve this relationship. If the multiple picozoan taxa did not group together, this gene should be considered to exclude from the concatenated dataset (because it is likely contaminated from different eukaryotes).
As mentioned above, gene trees from all genes used to build the phylogenomic dataset were manually inspected in several rounds of cleaning. It is worth mentioning that these trees were not only carefully inspected as part of this study after the addition of Picozoa (and a few other taxa), but the source dataset has been used numerous times in earlier publications from some authors on this study but also many others (most recently in: [Strassert JFH, Irisarri I, Williams TA, Burki F. 2021. A molecular timescale for eukaryote evolution with implications for the origin of red algal-derived plastids. Nat Commun 12:1879] or [Irisarri I, Strassert JFH, Burki F. 2021. Phylogenomic Insights into the Origin of Primary Plastids. Systematic Biol:syab036-]). Based on this long history of building and refinement, and our current meticulous effort to add Picozoa, we are confident that the presented dataset is of very high quality. More specifically, we found 252 genes containing multiple picozoan sequences from different SAGs that formed a clade in the initial single gene trees (i.e. before merging closely related taxa into OTUs to reduce missing data), not specifically related to any eukaryotic groups (as is expected for orphans like Picozoa), strongly suggesting that these are genuine picozoan sequences. In the minority of genes with a single retained Picozoa sequence, all discarded sequences could be clearly identified as contamination (because they were closely related to specific groups), or paralogs. We made the assumption that these few retained singletons were from Picozoa because they could not be clearly identified as belonging to any specific groups, despite our dense taxon sampling. However, as we describe below, the conclusions of our analyses remain the same even after discarding the genes containing these singletons. SAG33 is a special case that is not seen with any other SAG: many sequence fragments repeatedly branched together with a single cryptophyte species, *Teleaulax amphioxeia*, with nearly 100% sequence identity. Based on this observation, we concluded that SAG33 contained a high proportion of undigested cryptophyte DNA (or the other way around) and we decided not to include it in the final dataset. Moreover, as expected for this level of evolutionary divergence, very few single genes recovered the monophyly of Picozoa with red algae and rhodelphis (psmb3 and gdi2 and psma3 with only Cyanidioschyzon), all unsupported, although several genes showed monophyletic Picozoa and Rhodelphis (ARP2, crfg, eftud2, FCF1, KPNB1, mcm6, psmb6, rps3) or Picozoa and Rhodophyta (MAT1A, mcm2, NSA2, ODPB, PELO, psma1, psmb4, rpl14, rpl27a, RPS25, SEC23). This is a general observation that has become the rule in phylogenomic analyses of deep eukaryotic relationships: at the single-gene level the deep relationships are generally inconclusive due to lack of statistical support (this is for example also the case for Haptista or SAR). In order to resolve these deep nodes, including the Picozoa/Rhodelphis/Red algae clade, we needed to perform concatenation-based analyses, even though ASTRAL (that is based on a multispecies coalescent model) also recovers this topology; this analysis is now included in our manuscript (please see the ASTRAL description on p.5).

For another example, I was able to detect a Achromobacter 16S rDNA sequence with 100% identity and complete query coverage from ‘NODE_30_length_5267_cov_9249.629063 (position: 2,413 to 887)’ in the SAG02 assembly. Again, what if the authors included this kind of bacterial genes in the phylogenetic analysis, it would mislead the phylogeny. BlobTools (Laetsch & Blaxter, 2017) or Kraken2 (Wood et al., 2019) can be used to sort out viral or bacterial contaminations.
As discussed above, the assemblies are by no means clean of contamination (SAGs are generally “dirty” data), but the genes entering the phylogenomic dataset have been heavily curated. It is thus to be expected that contaminant sequences, such as the one the reviewer points out here, are found among the contigs. However, these data will not appear in the analyses because our phylogeny-aware manual approach to detect and remove contamination (and paralogy) from the

single-genes that we used in the phylogenomic analysis is the most powerful, and as such the (relatively low) level of contamination in the genome *assemblies* (not in the sequences entering the *alignments*) does not impact our robust results. This approach is described on p.12, first paragraph of the *phylogenomic* section.

BUSCO scores may be overestimated in this study due to some bacterial genes that are homologous to eukaryotic genes. BUSCO and CheckM (Parks et al., 2015) would be used for assessing the genome completeness of Picozoa. The authors simply combined partial and complete BUSCO genes, which I assume are mostly partial, to represent BUSCO in the manuscript. Furthermore, I was not able to find information about duplicated BUSCO genes, which would be required to assess their SAG genome accurately. Recovering complete picozoan genes is difficult due to methodological limitations of WGA, but readers should be made aware of this limitation.
To our knowledge, CheckM is unfortunately not applicable to eukaryotic genomes (as indicated in: [D. H. Parks, M. Imelfort, C. T. Skennerton, P. Hugenholtz, G. W. Tyson, CheckM: Assessing the quality of microbial genomes recovered from isolates, single cells, and metagenomes. Genome Research. 25, 1043–1055 (2015)]). We thank the reviewer for pointing out the lack of information for duplicated BUSCO genes, which we now provide as a new supplementary figure (Fig S13) including partial and duplicated BUSCO scores. If counting only complete BUSCOs the total completeness still reaches 63% (so there are only a few that are partial when looking at all SAGs/COSAGs), with only minimal duplicated BUSCOs (max 4%) per SAG/COSAG. We now also deposited the raw BUSCO results on FigShare (Picozoa: BUSCO results).

More importantly, the authors predicted Picozoa genes using prodigal, a prokaryotic genome prediction program, however, eukaryotic gene prediction needs to be done by considering intron splicing patterns. Because we don't know anything about introns in picozoan species yet, gene prediction should be done using tools that are typically used for eukaryotic gene modeling. BUSCO (with '-m geno' option) and AUGUSTUS (Stanke et al., 2006) with the training sets would be used to obtain gene structure information.
This is a good suggestion, one that we in fact actively worked on before the initial submission. We attempted to generate gene models using AUGUSTUS, Maker, Braker and Funannotate. Since there is no specific training data, we attempted to identify picozoan transcripts from oceanic metatranscriptomic datasets (Tara Oceans) and mapped those to our genome assemblies, but this approach proved unsuccessful. We also tried to use red algal and rhodelphis gene models, but this was also unsuccessful, likely due to the large evolutionary distances. Ultimately we determined that using Prodigal gene prediction, although not ideal, was the best workable solution which provided good gene predictions that we could verify by looking at the untrimmed single-gene alignments. For all phylogenomic markers, we checked alignments containing multiple picozoan sequences from the same contig and concatenated them if they were consecutive (for example
Picozoa_Picozoa_N/A_N/A_N/A_N/A_N/A_COSAG03@NODE_153_length_11455_cov_concat_8-5_plen_446 is the concatenation of proteins 8,7,6,5 from contig NODE_153_length_11455). An additional evidence that Prodigal works well to identify genes in Picozoa is the observation of mostly complete BUSCO scores. Overall, we prefer to keep our verified Prodigal predictions as we think that it provides a workable solution for the aims of this work, with no evidence that AUGUSTUS would outperform (and would require to run again *all* analyses with new gene models).

Furthermore, genome correction (e.g., Pilon) using Illumina raw data will reduce gene modeling errors by refining the genome assembly.
This is an interesting suggestion and a possibility that we would like to investigate in future studies. However, running Pilon now would change in effect every downstream analysis, which would mean several months of heavy computational time (and associated costs) utilizing dozens of cores of a computer cluster and, in our opinion, is likely to lead to little change to the end data set or insights gained from it. This is because we already performed read correction in Spades

and additionally ran MismatchCorrector using the --careful option, a post processing tool to minimize the number of mismatches in the final contigs.

Phylogenetic analysis

Although, the Picozoa have shown a strong monophyly with reds or Rhodelphis lineages, the strong monophyly supported in Archaeplastida or other lineages does not fit with conventional views, for example, the monophyly of Crytophyta and Rhodophyta (Fig. S3).

We are somewhat unsure of what the reviewer is referring to with "conventional views". This part of the eukaryotic tree is notable for a lack of consistency across studies, in particular the position of Cryptista, the monophyly of Archaeplastida, and the branching order within Archaepastida. In Figure S3, the cryptophyte nucleomorph sequences branch within red algae as expected, whereas the cryptistan nuclear sequences branch with Chloroplastida+Glaucophytes. This topology has been observed before (e.g. Burki et al. 2016), although it is by no means a stable topology; the position of Cryptista in the tree of eukaryotes remains unresolved, and we feel there is therefore no conventional view. Indeed, in several recently published papers (e.g. Strassert et al. 2021, Irisarri et al. 2021) and as shown in this manuscript when using better fitting models than in Fig S3 (see Figure 2), Cryptista tend to branch as sister to a monophyletic Archaeplastida, now also including Rhodelphis and Picozoa. We think that these relationships represent the best current hypotheses, but they will need to be tested and validated (or not) in the future.

Even though previous phylogenetic trees (Moreira & López-Garca, 2014) haven't showed high support values of picozoan lineage, an approximately unbiased (AU) test should be performed with all possible phylogenetic positions (especially to the topology of Archaeplastida without Picozoa). Therefore, I feel that those phylogenies were chosen by the author as a form of "cherry picking", because of their inconsistency with previous studies (Burki et al., 2012) and even the current study (Fig. S3).

As suggested by the reviewer, we have now performed the AU test with more competing topologies (now 15 in total) including Picozoa placed as sister to Archaeplastida, Cryptista and Telonemia as well as together with Cryptista within Archaeplastida; the results are shown in Table S4. Interestingly, all tested topologies were rejected except the tree where Picozoa branches sister to Rhodelphis+Rhodophyta (corresponding to the best ML and Bayesian tree) and the tree where Picozoa is most closely related to Rhodophyta (to the exclusion of Rhodelphis). In our view, these new analyses strengthen our main conclusion that Picozoa form a monophyletic grouping with Rhodelphis and Rhodophyta (which is also shown with the additional fast site removal, chi-square trimming and ASTRAL analyses–see below), although the exact branching order among these three lineages remains not fully certain. Please note also that some topological differences between the 794-taxa and the 67-taxa datasets are to be expected, since they e.g. use very different evolutionary models.

Because these results have the potential to alter our understanding of Archaeplastida evolution, I would suggest approaching this issue with a more conservative aspect. Contamination candidates or significantly divergent sequences that could mislead the phylogenetic signal should be deleted from the dataset. For example, using BMGE alignments from authors' dataset (from figshare 'Picozoa: 67 taxa alignments and trees'; 67 taxa, 317 genes), Fabomonas tropica in xpb phylogeny and Rhodelphis limneticus in BMS phylogeny (see figures below) showed relatively long branches compared to other eukaryotic lineages from gene trees reconstructed by IQ-TREE (-m TEST; 1K ultrafast bootstraps).

We thank the reviewer for the very thorough examination of the provided material. We verified the mentioned trees and alignments, and in both genes the trimmed alignments for these sequences happen to have very few positions left (see screenshots below). It has recently been shown that untrimmed alignments (or very gently trimmed) are more appropriate for reconstructing gene trees as they retain more phylogenetic signal; see for example: [1] Ali RH, Bogusz M, Whelan S. 2019. Identifying Clusters of High Confidence Homologies in Multiple Sequence Alignments. Mol Biol Evol 36:2340–2351; [2] Tan G, Muffato M, Ledergerber C, Herrero J, Goldman N, Gil M, Dessimoz C. 2015. Current Methods for Automated Filtering of Multiple Sequence Alignments Frequently

Worsen Single-Gene Phylogenetic Inference. Systematic Biol 64:778–791. Accordingly, we have used untrimmed alignments to produce the single-gene trees for our thorough quality checks of all phylogenomic markers (we applied trimming only prior to concatenation, for computational reasons). In those gene trees, the mentioned problematic sequences appear to behave normally (see screenshots below). We now provide all trees and the corresponding untrimmed alignments on the FigShare repository. Please find below the trees and trimmed alignment for the two genes mentioned by the reviewer, which show no clear issue of long branches or paralogy and highlight how little sequence was left after trimming in the species mentioned by the reviewer.

BMS1 tree based on **untrimmed** alignment:

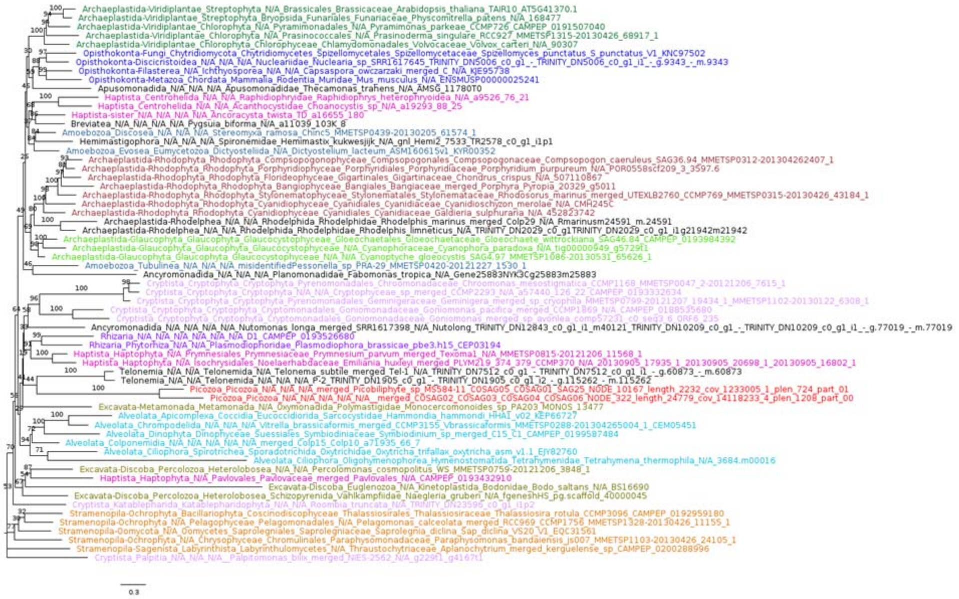

BMS1 BMGE **trimmed** alignment:

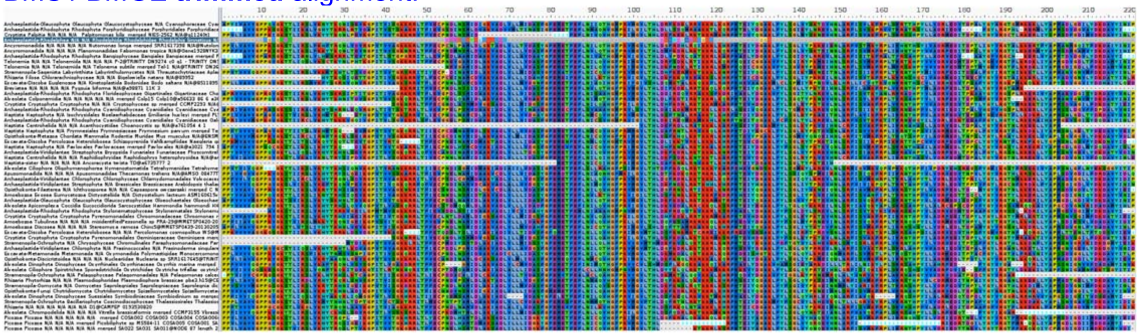

xpb tree based on **untrimmed** alignment:

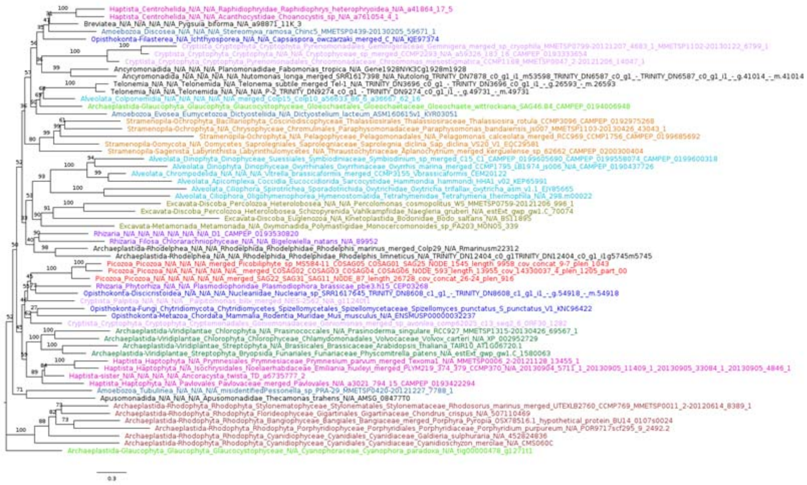

xpb BMGE **trimmed** alignment:

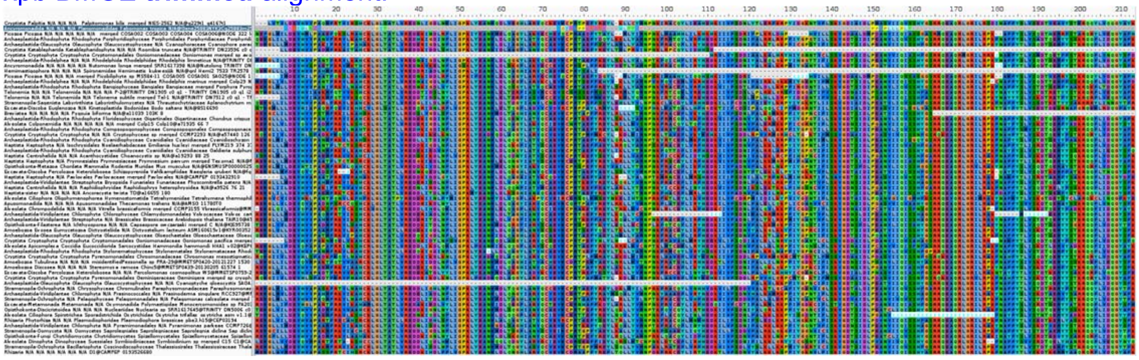

Additionally, I reconstructed the phylogenetic trees (from figshare 'Picozoa: cleaned single genes, initial supermatrix tree'; 760 taxa, 317 genes, midpoint rooting), and I was still able to find some paraphyletic signal, which might imply the contaminations or paralogs in their dataset (e.g., red algae paralogs marked with red color in the phylogeny of nhp2 as follows).

The issue of undetected paralogy or contamination is an important one in phylogenomics. We routinely perform very thorough checks of our alignments using automated (e.g. PREQUAL) and manual steps, and evaluate sequence homology based on manual curation of *all* single-gene trees which is the best possible way to do that. As already mentioned, the phylogenomic markers used in this study derive from previously published work that were also subjected to thorough curation, so that the source dataset prior to the new addition of Picozoa was already of very high-quality. Unfortunately, it is almost unavoidable that some minimal level of undetected paralogy errors remains in phylogenomic datasets, given the always larger size of these datasets and the manual steps required to clean the data. We have detected such errors in all our re-analyses of published phylogenomic datasets assembled by others, however it is very important to note that in the vast majority of cases correcting for these errors does not change the overall results. We thank the reviewer for detecting this case of paralogy in Rhodella in the nhp2 genes, this has now been corrected in the source dataset. In this particular case, this species was excluded from the final dataset and therefore did not influence the final results.

Furthermore, as mentioned earlier, picozoan genes used for this study should support monophyly of at least two picozoan species in a single gene tree. I found that 52 genes out of 317 have a single taxon of picozoa from the final dataset used in the main figure. To test whether these datasets support the current phylogenetic relationship, I would like to see, 1) a new phylogeny based on combined genes only with multiple picozoan taxa, and 2) combined gene set only with picozoan taxa (exclude genes without picozoan taxa). These reduced and conserved datasets would provide more accurate evolutionary history.

We thank the reviewer for suggesting these new analyses, which indeed would remove uncertainties related to the low numbers of picozoan sequences. The number of phylogenomic markers in our dataset with a single picozoan sequence (after removing contamination and paralogs) is in fact 24. All other genes containing Picozoa included sequences from more than one SAGs (from either the new SAGs or those previously available from Yoon et al. 2011). However, for the 67-taxon dataset, we collapsed closely related SAGs into OTUs (to reduce missing data), such that in the final dataset 52 genes indeed have a single picozoan OTU (which actually inflates the number of genes with one picozoan, since single-picozoan genes have multiple closely related SAG sequences collapsed into one). Out of the 317 markers, there are also 41 genes lacking any picozoa sequence. To meet with the reviewer's recommendation, we carried out one additional analysis including only the genes containing ≥ 2 picozoan OTUs, i.e. combining the two suggested analyses into one. This is because running these phylogenetic analyses based on such long alignments are computationally heavy, so we sought to address the issue while minimizing the computational cost. Accordingly, we ran a tree based on the concatenation after the removal of the 93 genes (52+41) containing only one or no Picozoa. This tree, which is reproduced below as well as in a new Figure S8, confirms the monophyletic relationship of Picozoa, Rhodelphis and Rhodophyta with maximal support. However, we note that this tree places Picozoa as the direct sister group to Rhodophyta (albeit unsupported), which is in agreement with the AU-test that did not reject this topology. We now mention in the text that although the monophyly of Picozoa, Rhodelphis, and Rhodophyta appears extremely robust, the branching order within these three main lineages is somehow less certain (on p.5: "*Although this group is robust, we observed one variation in the branching order between Picozoa, rhodelphids and red algae when trimming the 50% most heterogenous sites (Fig S7) and after removing genes with less than two picozoan sequences (Fig S8). In these analyses, Picozoa and red algae were most closely related, although this relationship was never significantly supported*").

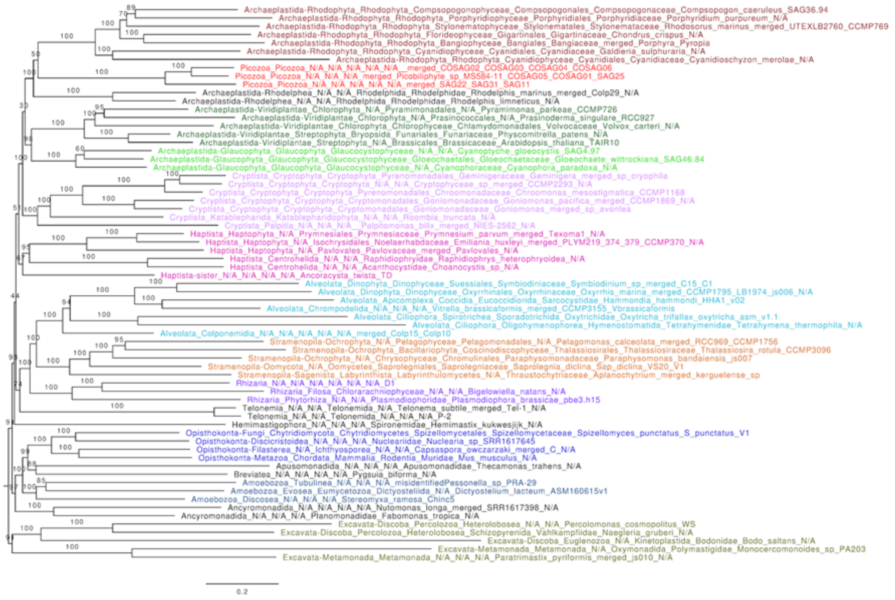

In page 8, "This search resulted in one protein (Arogenate dehydrogenase) with picozoan homologues that were closely related to red algae and belonged to a larger clade with host-derived plastid targeted plant sequences, but neither the picozoan nor the red algal sequences displayed predicted transit peptides." Provide phylogenetic tree regarding this gene.

The phylogenetic tree could be found on Figshare (Picozoa: plastid genes trees), but we erroneously failed to specify the name of the corresponding Orthologous group: OG0000831. We have now added this information to the main text (p.6, second paragraph).

I'm still skeptical of using concatenated data without serious validation step. Many phylogenetic studies have proven that just combing datasets does not always give a clear answer for species tree. For example, in this study, same dataset but different taxon sampling (794 taxa in Fig. S3) provided completely different phylogenetic relationship, especially Picozoa + Rhodelphia + Cryptopyta + Reds with strong bootstrap support (100%). To reach a correct species tree, I would suggest conducting other phylogenetic approaches (e.g., coalescence analysis - ASTRAL-III [Zhang et al., 2018], concordance tree - BUCKy [Ané et al., 2007]) as mentioned in these studies (Lemmon and Lemmon, 2013; Simmons & Gatesy, 2015). In addition, as found in recent study (Irisarri et al., 2021), very careful data and phylogeny validation were conducted (e.g., random sample of amino acid positions, quantifying gene support, normalized Robinson-Foulds distance, novel alignment descriptive metrics), which I was expecting in this current study.
First we note that although concatenation analysis is sometimes criticized, it remains the approach of choice and standard (best) practice for deep phylogenomic analysis (e.g. at the supergroup level). The choice of methods should be informed by the largest sources of error. Most importantly, at shallower timescales gene trees can be accurately inferred and for example ILS (which ASTRAL can take into account) can be a large source of variance among gene trees. However, at deeper timescales such as for the inference of the position of Picozoa, we think that sources of errors such as ILS become relatively less important and many other processes intervene to make the inference of individual gene trees much harder (and thus the rationale for running coalescence analysis becomes unclear). A nice recent discussion of these issues can be found in this review: David Bryant, Matthew W. Hahn. The Concatenation Question. In: Phylogenetics in the Genomic Era, pp.3.4:1--3.4:23, 2020. Second, it is fully expected that the different datasets in our study, which were analysed under very different models of evolution (e.g. site-homogeneous vs site-heterogeneous), give different topologies, so this should not be interpreted as evidence for conflicting signals. That said, we now provide a range of new analyses based on different alterations of our dataset to hopefully address the reviewer's concerns. Despite our relative skepticism described above, we have performed a tree reconstruction using ASTRAL-III, which is compatible under the multi-species coalescent model taking into account variability at the single-gene level. In this analysis, the position of Picozoa was fully consistent with the supermatrix analyses, which were placed in a monophyletic group together with Rhodelphis and Rhodophyta and strongly supported based on both ASTRAL posterior probabilities and multi-gene bootstraps (Figure S6). We additionally performed fast-site removal analyses to remove homoplasy and here too the Picozoa/Rhodelphis/Rhodophyta clade was robustly recovered, up until too much of the data was removed when the support starts to crumble (Figure S4). Finally, the monophyly of these three groups was also robust to chi-square trimming of the alignment to account for possible amino acid compositional bias (with both 25% (Fig S5) and 50% (Fig S7) of most heterogeneous sites removed), although the topology shifted towards Picozoa as sister to Rhodophyta with the more aggressively chi-square trimmed alignment (Figure S7), as already observed as a possibility in the AU-test and after retaining only the genes with ≥ 2 picozoan OTUs (see above). We think that these additional analyses described on p.5 strengthen our conclusion of a new large group of eukaryotes including Picozoa, Rhodelphis, and Rhodophyta, but that again the specific relationships within that group will need to be addressed in the future with more data.

Along with these suggestions, I am curious about the phylogeny using mitochondrial genome data, because the authors completed (or nearly completed) five mitochondrial genomes. Janouškovec, J. et al. (2017) already provided mitochondrial genome phylogeny with relatively strong bootstrap support values among major eukaryotic groups. If the authors provide a phylogeny using mitochondrial genomes data, then it will be one good evidence to support their hypothesis.
This is an interesting suggestion that we have attempted to address. The mentioned dataset from Janouškovec et al. in fact includes mostly nuclear-encoded mitochondrial proteins, so to utilize our new picozoan mitogenomes, we extracted the 10 mitochondrial genome-encoded proteins (atp6, cob, cox1, cox2, cox3, nad, nad3, nad4, nad4L, nad5) to run a phylogeny after adding Picozoa. The resulting phylogeny shows Picozoa branching with red algae and other cryptists, but it is very poorly resolved, which is not unexpected given the short length of this alignment and the generally poor signal in mitochondrial phylogeny for deep relationships. We show the tree below for the

reviewer's interest, but based on this tree we don't see the real value of presenting it in the manuscript, given that we already have a considerable quantity of supplementary material, and that all presented analyses converge towards the same solution as discussed in this letter.

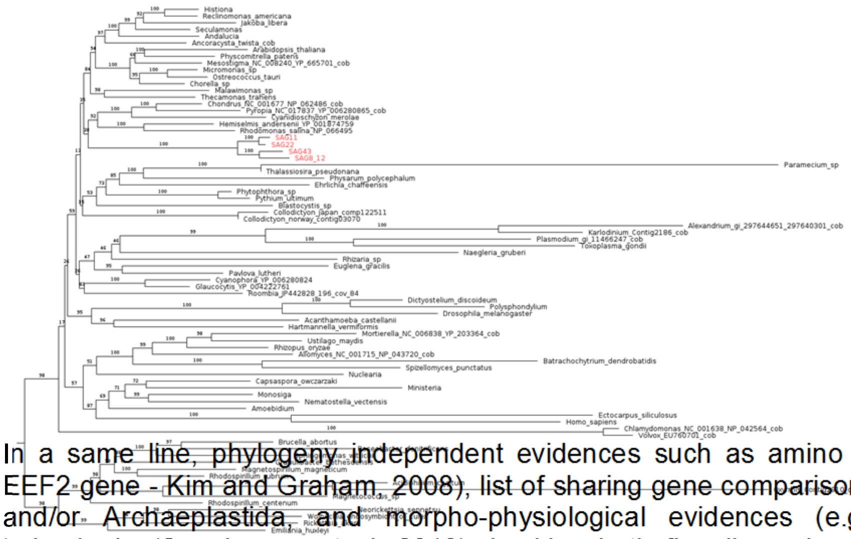

In a same line, phylogeny independent evidences such as amino acid insertion/deletion (e.g., EEF2 gene - Kim and Graham, 2008), list of sharing gene comparison between Picozoa, red algal and/or Archaeplastida, and morpho-physiological evidences (e.g., TEM) from Picomonas judraskeda (Seenivasan et al. 2013). Lacking both flagella and centriole are unique red algal characters, and these related genes would be good candidate to present.
We thank the reviewer for the suggestion of checking the EEF2 gene for the two amino acids replacement signature proposed in the listed paper. We identified in Picozoa (and Rhodelphis) the same consecutive SA residues that were previously shown in Rhodophyta, Chloroplastida, Cryptophytes, and Haptophytes and proposed to have occurred as a replacement of the ancestral GS residues. The presence of SA in Picozoa supports their affiliation with Rhodophyta. We now mention this in the text (on p.5) and show the alignment for EEF2 in Supplementary Data S1. As for other phylogeny-independent characters, we did not find obvious morphological synapomorphies between the described picozoan species and red algae nor Rhodelphis. Picozoa and Rhodelphis both possess flagella, so that the lack of flagella and centriole in red algae is most likely the result of a loss event after the divergence of the last red-algal common ancestor. Similar to Rhodelphis, we could identify homologs of 194 flagellar-associated proteins of the 361 Chlamydomonas reinhardtii proteins that were labelled as 'high confidence' based on the mass spectrometry data of [1. G. J. Pazour, N. Agrin, J. Leszyk, G. B. Witman, Proteomic analysis of a eukaryotic cilium. *J Cell Biol*. **170**, 103–113 (2005), 2. http://chlamyfp.org/index.php].

Overall, limited taxon sampling, contamination or paralogs issues (which need to be addressed manually), and well-supported conflicts among trees (e.g., Fig. 2 vs. Fig. S3) continue to be major concerns that weaken the results presented in this paper and bring into question its major conclusion.
As discussed throughout this response letter, we think that conflicts among trees that are based on vastly different taxon-sampling and–most importantly–very different models of evolution (relatively fast but less accurate models for the large dataset versus much more complex models for the reduced taxon sampling) are to be expected and accordingly commonly occur in similar phylogenomic studies. Despite these common conflicts, the strong argument that we make is based on determining the statistical fit of models and weighting more heavily the trees that are derived from the best fitted models (site heterogeneous CAT+GTR+G and LG+C60+F+G+PMSF models). We stress that when taking all our results together, a very consistent and extremely well supported picture emerges that shows the monophyletic relationship of Picozoa with Rhodelphis and Rhodophyta; these relationships are actually consistent in Figures 2 and S3). We also would like to state again that we present a very high-quality dataset containing to our knowledge the largest gene and taxon sampling to date for phylogenomic analysis (317 genes, 794 taxa), that has gone through the best practice of quality checks in the field of phylogenomics. All genes were

automatically and manually inspected numerous times based on superior gene trees for issues such as contamination and paralogy. Based on these checks, we think that the orthology of all sequences that were retained in the final markers has been evaluated to the best of our abilities. This does not prevent a few undetected paralogy (or contaminant) events, as identified by the reviewer, because unfortunately there is no robust fully automated way to screen hundreds of genes. Very recently, a method paper on best phylogenomic practices (which we have contributed to establish) presented a package of tools to assemble large datasets [Tice AK, Žihala D, Pánek T, Jones RE, Salomaki ED, Nenarokov S, et al. (2021) PhyloFisher: A phylogenomic package for resolving eukaryotic relationships. PLoS Biol 19(8): e3001365]. Despite several new scripts, the authors of this paper, several of which have been using phylogenomics for more than a decade, insist on the importance of the visual (manual) inspection of the trees to identify and delete paralogous sequences. We fully adhere to this practice, which makes the assembly of high-quality phylogenomic datasets cumbersome but at the same time allows (rarely) for paralogy to remain undetected. Most importantly however, based on the consistency of our results, we believe that our results are extremely robust to these few potentially undetected cases of paralogy.

Reviewer #2 (Remarks to the Author):

This article presents the analysis of 43 single-cell genomes of the poorly-known heterotrophic protist group Picozoa. Phylogenetic analyses based on a large concatenation of conserved proteins support that Picozoa branch within the Archaeplastida, as sister group to red algae and the phagotrophic rhodelphids. Nevertheless, despite this phylogenetic position, the authors did not find any clear genomic evidence for the present or past presence of plastids in these organisms. These are unexpected results that suggest that Picozoa may be the first known case of a completely plastid-lacking lineage within the Archaeplastida.

I think that these are very interesting results and I only have relatively minor comments.
We like to thank the reviewer for the very positive and constructive feedback.

- Line 97. It would be useful that the authors give a short explanation of what is “Pico-PCR”.
We updated the sentence to say “PCR with Picozoa-specific primers”, as described in Seenivasan et al. 2013.

- Line 98: “The sequencing reads were assembled into genomic contigs, ranging in length from 350 kbp to 66 Mbp”. Does it mean that all contigs were >350 kbp, up to 66 Mbp? Or do you refer to the total sum of contig sizes? It is unclear.
Sorry for the confusion. We meant the total sum of contig sizes, and updated the sentence on p.3 accordingly: “with total assembly sizes ranging from 350 kbp to 66 Mbp”

- Line 115: “The deep-branching picozoan lineages identified by Moreira and López-García (2014), as well as other possibly early-diverging lineages were not represented in our data”. The delimitation of lineages is always arbitrary but I think that the groups ‘BP2’ and ‘Deep branching 1’ could be considered to form a single group (including also SAG11). In that case, some SAGs would be ‘Deep branching 1’ representatives.
We agree with the reviewer that these lineage delimitations are arbitrary and should probably be updated in a future study of Picozoa diversity, but we included them here in order to be able to easily compare with older publications.

- Line 168: “An approximately unbiased (AU) test rejected the topology where Picozoa branched with rhodelphids (p=0.0362), but the position of Picozoa as the closest sister to red algae could not be rejected (p=0.236)”. Did the authors do a similar test with Picozoa as sister group to Archaeplastida? I think it is an important possibility that has to be tested too. Testing topologies

where Cryptista branch within Archaeplastida (as it has been reported in previous articles) can also be very interesting with this dataset enriched in picozoan taxa.
As described in details in response to R1, we have repeated the AU-test with more topologies (15 in total), including as suggested by the reviewer Picozoa as sister to Archaeplastida and Cryptophyta, and both together within Archaeplastida (see text on p.5 and Supplementary Table 4). All but the two previously not rejected topologies were rejected in this new analysis.

- In relation with the latter point, this region of the phylogeny of eukaryotes has been very unstable in recent years and here the authors present a tree that is, once again, very different from previous ones. Therefore, it is important to be cautious and provide as much evidence as possible. For example, it would be very interesting to show the result of a slow-fast analysis to see if fast evolving sites influence this tree topology.
We agree with the reviewer that this region of the tree is notoriously unstable. We thank the reviewer for the suggested analysis, which we performed in the way of a fast-site removal analysis (new Fig S4). In this new analysis, the Picozoa/Rhodelphis/Rhodophyta clade remained robust until too much of that data was removed (after about 64% of the alignment sites were removed). In addition, we also tested for possible biases due to AA compositional heterogeneity. In the original analysis, we used the CAT+GTR+G model to account for across-site compositional heterogeneity. In the revised manuscript, we have also performed analyses in which we removed the top 25% and 50% of sites that contribute to branch heterogeneity. This was done with chi-square trimming to evaluate the potential negative impact of compositional biases that could still result in model misspecification in spite of using the best-fitting CAT+GTR+G model. Here too, the monophyly of Picozoa/Rhodelphis/Rhodophytas remains robust, although the topology shifted towards Picozoa as sister to Rhodophyta with the more aggressively chi-square trimmed alignment (main text on p.5 and Figs S5 and S7).

- Line 252: “This position has important implications for our understanding of plastid origins because, in contrast to all other archaeplastids known to date, our results strongly indicate that Picozoa lack plastids and plastid-associated EGTs”. I think this sentence is too strong, the authors found some candidates and, despite the EGT/HGT ratio, we cannot completely exclude that they represent real EGT cases.
Fair point. We agree that the possibility that the reported few cases are real EGT cannot be excluded. Accordingly, we removed the adverb ‘strongly’ from the sentence in order to be more open to the alternative hypothesis.

- Line 267: “A related explanation could involve a secondary endosymbiosis where the plastid in red algae, for example, was secondarily acquired from a green alga. The latter scenario would be effectively refuted by the identification of host-derived plastid components shared between all archaeplastid lineages”. If the hosts are closely related, can you imagine to observe ”shared” host-derived plastid components derived from convergent acquisitions?.
This is indeed a theoretical possibility, but one that we think would be very unlikely because it would have to involve the independent de novo evolution, or independent repurposing, of homologous host proteins to function in the plastids across the archaeplastid lineages. To tone down this sentence, we changed it to (p.8): “This latter scenario would be made unlikely by the identification of host-derived plastid components shared between all archaeplastid lineages.”

Reviewers' Comments:

Reviewer #1:
Remarks to the Author:
I found the revised version adequately addressed most of comments that I raised. Since different options resulted different phylogenetic relationships, I would suggest presenting an alternative evolutionary scenarios in the main text.

Reviewer #2:
Remarks to the Author:
After reading the new version of the article and the rebuttal to the Reviewers' comments, I think that the authors have made a good job to address all of the criticisms. Although I am quite sure that this will not be the final answer for this part of the tree of eukaryotes, I think that this paper makes a very interesting contribution and I support its publication.

REVIEWERS' COMMENTS

Reviewer #1 (Remarks to the Author):

I found the revised version adequately addressed most of comments that I raised. Since different options resulted different phylogenetic relationships, I would suggest presenting an alternative evolutionary scenario in the main text.

Thank you very much! We have addressed the suggestion of the reviewer by presenting a modified Figure 2 that now includes an insert of the only other topology that we have recovered for the position of Picozoa (so Figure 2 now includes both topologies). We think that this is the most transparent solution to present the recovered alternatives. To summarize our main conclusions regarding the phylogenetic origin of Picozoa, our analyses show that (1) Picozoa is related to red algae, this is statistically strongly supported in all our analyses, and (2) the most favored topology is that Picozoa is sister to both red algae and Rhodelphis, but we cannot fully exclude that Picozoa is most closely related to red algae (to the exclusion of Rhodelphis). To acknowledge that this uncertainty can impact our interpretation of plastid evolution–specifically the scenario of independent endosymbioses–we added a mention to *Rhodelphis* in the following sentence (here underlined) appearing in the discussion (2nd paragraph): "To suggest that Picozoa was never photosynthetic requires that the current distribution of primary plastids is due to multiple independent endosymbioses, specifically that red algae (and possibly *Rhodelphis*) arose from one or two separate primary endosymbiosis from that leading to green algae and glaucophytes".

Reviewer #2 (Remarks to the Author):

After reading the new version of the article and the rebuttal to the Reviewers' comments, I think that the authors have made a good job to address all of the criticisms. Although I am quite sure that this will not be the final answer for this part of the tree of eukaryotes, I think that this paper makes a very interesting contribution and I support its publication.

Thank you very much! We fully agree that this result might change in the future with e.g. the discovery of further archaeplastidal lineages.
